# Marine Biodiversity of *Aotearoa* New Zealand

**DOI:** 10.1371/journal.pone.0010905

**Published:** 2010-08-02

**Authors:** Dennis P. Gordon, Jennifer Beaumont, Alison MacDiarmid, Donald A. Robertson, Shane T. Ahyong

**Affiliations:** National Institute of Water and Atmospheric Research, Kilbirnie, Wellington, New Zealand; University of Canterbury, New Zealand

## Abstract

The marine-biodiversity assessment of New Zealand (*Aotearoa* as known to Māori) is confined to the 200 nautical-mile boundary of the Exclusive Economic Zone, which, at 4.2 million km^2^, is one of the largest in the world. It spans 30° of latitude and includes a high diversity of seafloor relief, including a trench 10 km deep. Much of this region remains unexplored biologically, especially the 50% of the EEZ deeper than 2,000 m. Knowledge of the marine biota is based on more than 200 years of marine exploration in the region. The major oceanographic data repository is the National Institute of Water and Atmospheric Research (NIWA), which is involved in several Census of Marine Life field projects and is the location of the Southwestern Pacific Regional OBIS Node; NIWA is also data manager and custodian for fisheries research data owned by the Ministry of Fisheries. Related data sources cover alien species, environmental measures, and historical information. Museum collections in New Zealand hold more than 800,000 registered lots representing several million specimens. During the past decade, 220 taxonomic specialists (85 marine) from 18 countries have been engaged in a project to review New Zealand's entire biodiversity. The above-mentioned marine information sources, published literature, and reports were scrutinized to give the results summarized here for the first time (current to 2010), including data on endemism and invasive species. There are 17,135 living species in the EEZ. This diversity includes 4,315 known undescribed species in collections. Species diversity for the most intensively studied phylum-level taxa (Porifera, Cnidaria, Mollusca, Brachiopoda, Bryozoa, Kinorhyncha, Echinodermata, Chordata) is more or less equivalent to that in the ERMS (European Register of Marine Species) region, which is 5.5 times larger in area than the New Zealand EEZ. The implication is that, when all other New Zealand phyla are equally well studied, total marine diversity in the EEZ may be expected to equal that in the ERMS region. This equivalence invites testable hypotheses to explain it. There are 177 naturalized alien species in New Zealand coastal waters, mostly in ports and harbours. Marine-taxonomic expertise in New Zealand covers a broad number of taxa but is, proportionately, at or near its lowest level since the Second World War. Nevertheless, collections are well supported by funding and are continually added to. Threats and protection measures concerning New Zealand's marine biodiversity are commented on, along with potential and priorities for future research.

## Introduction

**Figure 1:**
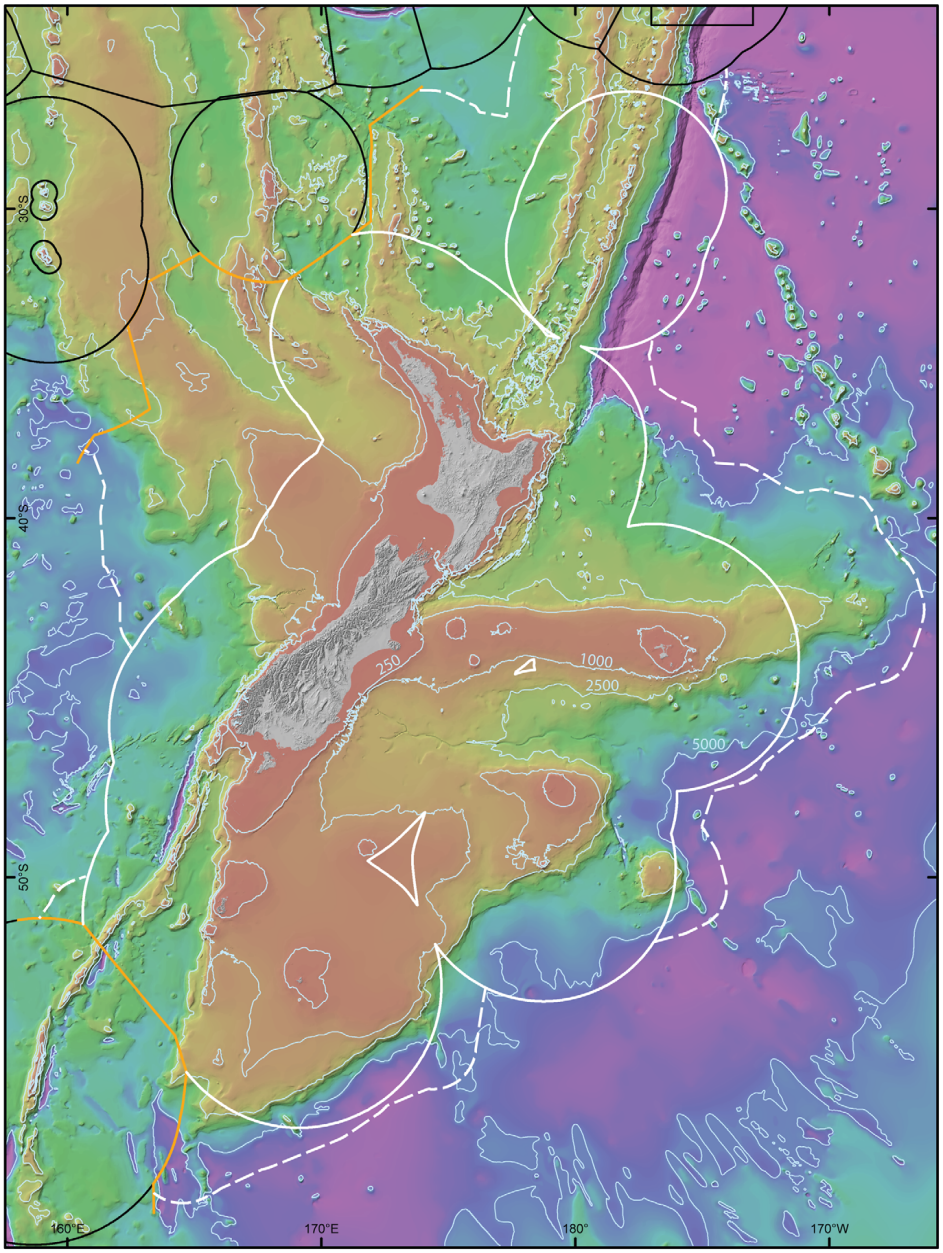
The New Zealand region continental mass (Zealandia), seafloor, and Exclusive Economic Zone. Bathymetric contours indicate 250, 1,000, 2,500, and 5000 m. The solid white line shows the boundary of the New Zealand EEZ, the solid black line the EEZs of Australia and island states to the north; the dashed white line indicates the extension to New Zealand's legal continental shelf, and the orange line the agreed Australia/New Zealand boundary under UNCLOS.

*Aotearoa* New Zealand is the most oceanic nation of significant size, in the world's largest ocean. Its western coast is 1,600–2,250 km from Australia; it has a land and freshwater area of 268,680 km^2^, and an Exclusive Economic Zone (EEZ) of almost 4.2million km^2^ that spans 30 degrees of latitude and exceeds 15 times the land area (Figure 1). This sea area, large in relation to the majority of the EEZs of the Pacific states (Table 1), constitutes both a huge resource reservoir and a major management and biodiversity-exploration challenge to a country with a population of only 4.3 million people.

**Table 1:** The marine-biodiversity and data-management challenge in the Pacific Ocean―size of EEZs in relation to population base and land area.

State/Territory	Land area km^2^	EEZ area ×1,000 km^2^	EEZ vs land area (times larger, rounded)	Population July 2009 estimate*
American Samoa	197	390	1,980	65,628
Cook Islands	180	1,830	10,167	11,870
Fiji	18,376	1,290	70	944,720
French Polynesia	3,521	5,030	1,429	287,032
Guam	549	218	397	178,340
Kiribati	727	3,550	4,883	112,850
Marshall Islands	720	2,131	2,960	64,522
Micronesia (Fed. States)	702	2,978	4,242	107,434
Nauru	21	320	15,238	14,019
New Caledonia	19,103	1,740	91	227,436
New Zealand	268,200	4,199	16	4,305,150
Niue	258	390	1,512	1,398
Northern Marianas	475	1,823	3,838	88,662
Palau	500	629	1,258	20,796
Papua New Guinea	461,690	3,120	7	6,057,263
Pitcairn	5	800	160,000	48
Samoa	2,934	120	41	219,998
Solomon Islands	29,785	1,340	45	595,613
Tokelau	12	290	24,167	1,416
Tonga	696	700	1,006	120,898
Tuvalu	26	900	34,615	12,373
Vanuatu	12,189	680	56	218,519
Wallis and Futuna	124	300	2,419	15,289
**Total**	**820,989**	**34,768**		**13,671,274**

https://www.cia.gov/library/publications/the-world-factbook/rankorder/2119rank.html (accessed 2009 May 29).

Source: Adapted from SPREP [84].

New Zealand is an archipelago comprising two major islands and more than 700 offshore islands and islets. Most of the islands are small and lie within 50 km of the coast. Collectively, the mainland and smaller islands of New Zealand are the visible surface of a large submerged continental mass that extends beyond the boundaries of the EEZ. This landmass, geologically known as Zealandia, extends from New Caledonia's Chesterfield Plateau (about 19° S) in the northwest and the Colville and Kermadec Ridges in the northeast to south of New Zealand's sub-Antarctic islands at about 56° S. The combined seafloor area of the plateaus and ridges is about 6 million km^2^; the emergent land is only about 7% of the area of continental crust represented by Zealandia [1]. The area of shallow shelf is about 300,000 km^2^, but the legal concept of an “extended continental shelf” encompasses the whole area underlain by continental crust and includes the shelf and slope. Hence, New Zealand's extended continental shelf is potentially 1.7 million km^2^. Half the area of the existing EEZ is shallower than 2,000 m (Table 2). The latitudinal spread of New Zealand, coupled with the varied seafloor relief, is accordingly mirrored by the wide diversity of marine habitats [2].

**Table 2:** Relative proportions of depth ranges in the New Zealand region.

Depth range (m)	New Zealand region (%)	New Zealand EEZ (%)
0–1000	12	28
1000–2000	13	21
2000–3000	11	15
3000–4000	12	12
4000–5000	25	16
5000–6000	26	6
6000–7000	0.5	1
7000–8000	0.5	0.5
8000+	0.2	0.5

Note: The New Zealand region comprises about 2.7% of the world seafloor, bounded by 24°–57°30′ S, 157° E–167° W, and EEZ (4.2 million km^2^). From Nelson & Gordon [85].

The New Zealand EEZ, possibly the fifth-largest in the world after those of the United States, Australia, Russian Federation, and French Polynesia, owes its large size to the widely distributed emergent outer islands of Zealandia. Hence, subtropical Raoul Island, at 29°15' S on the Kermadec Ridge, is about 1,000 km from the North Island coast, the Chatham Islands are 800 km east of South Island, and the farthest sub-Antarctic island — Campbell Island — is 640 km south of South Island at 52°30′ S.

The New Zealand coastline is very long. Estimates range from 15,000 to 18,000 km, the exact length being confounded by the fractal quality of inlets, headlands, spits, bays, harbors, fiords, sounds, and estuaries. Although the North Island is smaller in area than the South Island, it has a longer coastline owing to extensive embayments. Beyond the shores, the seafloor slopes relatively gently to the shelf break at about 100–200 m depth. This relatively shallow shelf extends for only a few hundred meters off Fiordland, but is up to 250 km wide in western Cook Strait and beyond [2].

As conceptualized by the U.S. National Oceanic and Atmospheric Administration, the shallow shelf area corresponds to the New Zealand Large Marine Ecosystem (LME no. 46 out of 64 globally), LMEs being defined as relatively large areas of ocean space of approximately 200,000 km^2^ or greater, adjacent to the continents in coastal waters where primary productivity is generally higher than in open ocean areas. Such formulations are not very helpful in the case of New Zealand, which is oceanographically highly diverse and reviews of the LME [3] are overly simplistic and misleading. The same area also more or less corresponds to the World Wildlife Fund so-called Global 200 New Zealand Marine Ecoregion, although for recent analyses of this region, the entire EEZ has served as a larger proxy [4], [5].

### Geological history, physical, geological, oceanographic and biological setting

New Zealand's geological history, temporal and geographical isolation, and physiographical complexity have helped shape the character of its marine biodiversity. Zealandia began to split away from Australia and Antarctica 100 million to 83 million years ago (Ma) in the early Late Cretaceous. During the Cenozoic, there were large variations in archipelago structure owing to fluctuations in sea level and tectonic activity and rotational displacements of the landmass. By 23 Ma, at the end of the Oligocene, Zealandia is believed to have become nearly or totally submerged [6], with consequent effects on subsequent repopulation of the land, as well as the evolution of the shelf biota at that time in the absence or near absence of land-derived sediments and nutrients. By 5 Ma, the Southern Alps were a continuous mountain chain yielding abundant sediment to the shelf and deep sea. Continuous mountain building, earthquakes, and volcanism caused by plate movements throughout the Neogene contributed to Zealandia's present configuration. The Pleistocene glaciations, covering the past 2.6 million years, have been considered important in determining the character of much of New Zealand's present-day shelf biota. Over the past 10,000 years, uplift of the Southern Alps has continued at an average of 10 mm or more a year. It is offset by erosion, which results in huge quantities of sediment being transported to both coasts, onto the shelf, and into the deep sea via canyons and channels. Currently, the mountains of New Zealand contribute a massive 209 million metric tons of sediment annually to the sea (1% of the mud supply to the world's oceans from less than 0.2% of its land area) [7]–[9]. These erosion rates, exacerbated by extensive removal of native-forest cover, have led to significant carbon transfers and losses (about 4 million metric tons of carbon per year) to the ocean [10].

Zealandia is cut diagonally from northeast to southwest by the boundary between the Pacific crustal plate to the east and the Australian plate to the west. This boundary is associated with the northeast-trending 10,047 m deep Kermadec Trench, volcanic Kermadec Ridge — part of the Pacific “Ring of Fire” — and associated back-arc basin. On land, the plate boundary passes along the alpine fault and continues on to the southwest, paralleling Puysegur Bank and Trench (up to 6,200 m deep) and the northern part of the Macquarie Ridge Complex. Zealandia is thus still active tectonically, with areas of uplift and subsidence, and onshore and offshore volcanism. The line of mainly submerged volcanic cones along the western side the Kermadec Ridge runs southwards to the mainland shelf. Hydrothermal vents that occur on some of the volcanoes [11]–[13] in this ridge-trough area support chemosynthetic ecosystems [14]. Off the mainland coast, shelf-edge instability is enhanced in some locations by cold seeps of methane-rich fluids that likewise support chemosynthetic faunas and carbonate concretions [15]–[17]. Data on seamount features in the region are summarized in Table 3.

**Table 3:** NIWA data on seamount features in the New Zealand EEZ and wider region.

Depth at summit	<750 m	750–1,249	1,250–2,499 m	2,500+ m	Total
No. in EEZ	153	225	225	115	718
Seamount area in EEZ (km^2^)	10,169	44,719	32,388	25,440	112,716
No. fished – 5 or more tows	51	106	4	0	161
No. outside EEZ	59	59	82	295	495

Note: Seamounts are defined as having a vertical elevation of >100 m.

**Figure 2:**
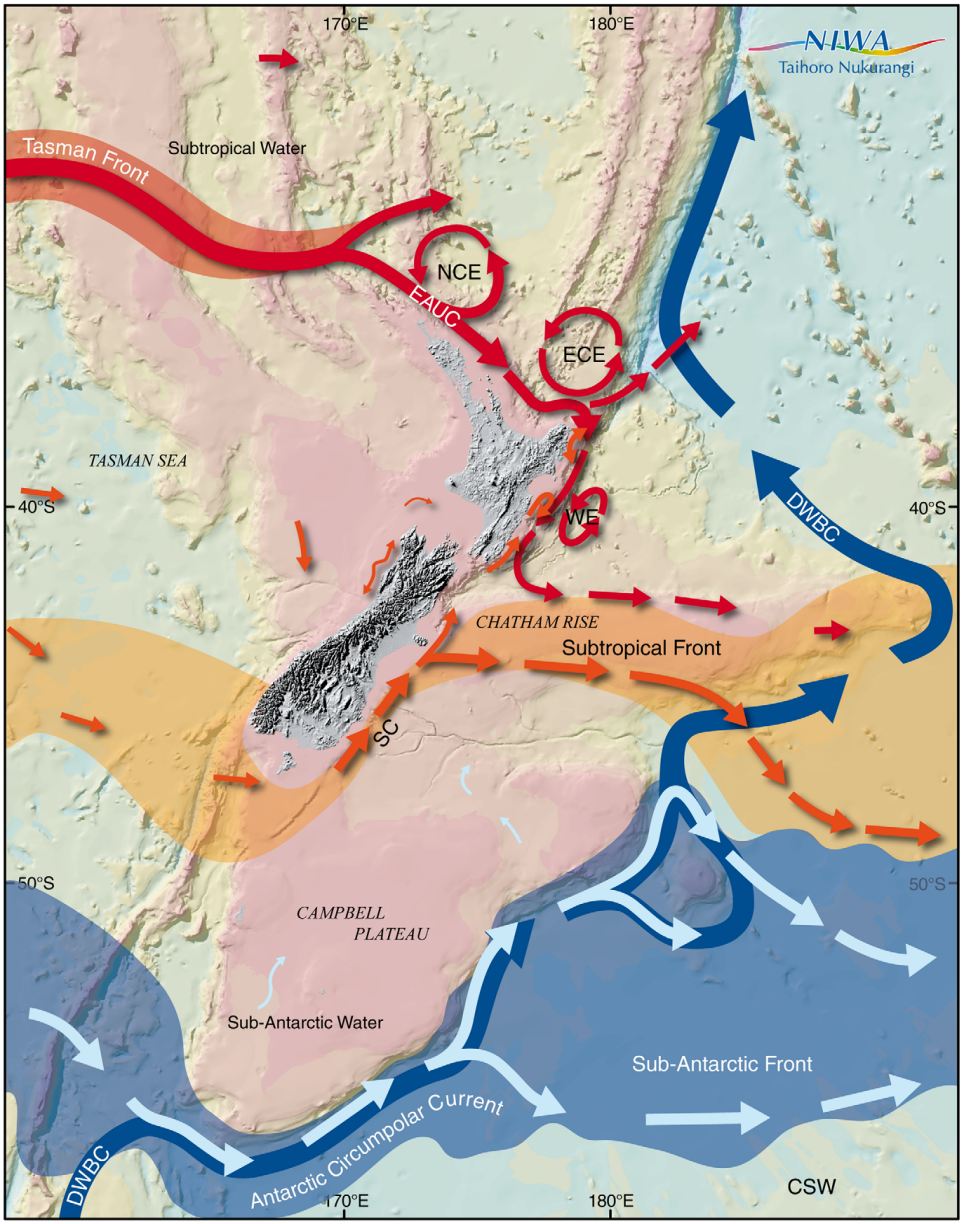
Circulation in the New Zealand region, showing the major fronts and eddy features. EAUC, East Auckland Current; ECC, East Cape Current; NCC, North Cape Current; SC, Southland Current; WE, Wairarapa Eddy; DWBC, Deep Western Boundary Current.

The New Zealand archipelago forms the western boundary to the South Pacific Ocean south of 34°. The country thus influences the flow of the major water masses and results in shelf-edge currents and oceanic eddies that interact with coastal waters over the shelf, bringing oceanic water into the coastal zone. Insofar as the New Zealand landmass interrupts what would otherwise be a mainly zonal flow, it effectively splits it, such that, in general terms, the water flows in a clockwise direction around the northern half and anticlockwise around the southern half of New Zealand, and the merged flow continues eastward. Overall, New Zealand intercepts from the west two major surface-water masses, which have distinctive temperature-salinity characteristics [18]. Boundaries between water masses define major thermohaline fronts, which are generally synonymous with ocean currents. These are shown in Figure 2. The tidal regime around New Zealand is semidiurnal. New Zealand acts as an amphidromic node in that a trapped wave rotates around the main landmass in a counterclockwise direction. The tidal range is 1–2 m.

The nutrient and chemical environment in the seas around New Zealand has been summarized by Bradford-Grieve et al. [19]. The seasonal succession of phytoplankton is typical of temperate waters [20]. Mean oceanic phytoplankton biomass is unremarkable in a global context.

### History of research and species discovery

The history of marine exploration in New Zealand was summarized in the 1970s [22]. The first explorations that yielded a useful quantity of specimens and data for scientific study were those of James Cook in 1769 and 1773–74 and of Louis Duperry and Dumont D'Urville in 1824, 1827, and 1840. Many fishes, mollusks, crustaceans, and other invertebrates were discovered and named from these voyages. Between 1835 and 1850, four of the most famous naturalists of the nineteenth century visited New Zealand — Charles Darwin, J. D. Hooker, T. H. Huxley, and James D. Dana, each of whom contributed to the knowledge of the marine biology of the country. Other smaller expeditions, and the reports of their voyages, are also mentioned by Putnam [22], who additionally described the work of the new group of resident naturalists that made New Zealand their home after the 1840s. With the founding of several scientific societies, provincial museums, and university colleges during the second half of the nineteenth century, the scientific study of New Zealand's marine biology and physical environment was assured. It was during this period, in 1874, that the expedition of HMS *Challenger* made the first deep-sea oceanographic observations in New Zealand waters, taking surface-to-bottom sea temperatures and sampling bottom sediments and marine life [23]. The Danish *Galathea* expedition collected in the area in 1952 [24].

### Census projects and regional OBIS Node

Census of Marine Life field projects involving New Zealand scientists include Global Census of Marine Life on Seamounts (coordinated from the National Institute of Water and Atmospheric Research – NIWA), Biogeography of Deep-Water Chemosynthetic Ecosystems, Census of Antarctic Marine Life, International Census of Marine Microbes, Continental Margin Ecosystems, Census of Diversity of Abyssal Marine Life, History of Marine Animal Populations, Tagging of Pacific Predators and, peripherally, Census of Marine Zooplankton. New Zealand is the focal point of the Southwestern Pacific Regional OBIS Node (http://nzobis.niwa.co.nz/), hosted by NIWA in Wellington. Subsets of the data held by this node have been provided to the OBIS central portal at Rutgers University, and from there to the Global Biodiversity Information facility in Copenhagen. These data can be downloaded directly, or specific subsets that may be more suitable for analyses addressing particular research questions can be accessed from other databases held by NIWA. Many of the distributional records of the more common marine species are also available as synoptic distribution maps on the National Aquatic Biodiversity Information System, which is a service provided to the Ministry of Fisheries by NIWA at http://www2.nabis.govt.nz/Map.aspx.

## Materials and Methods

### Biological-oceanographic and fisheries data

**Figure 3:**
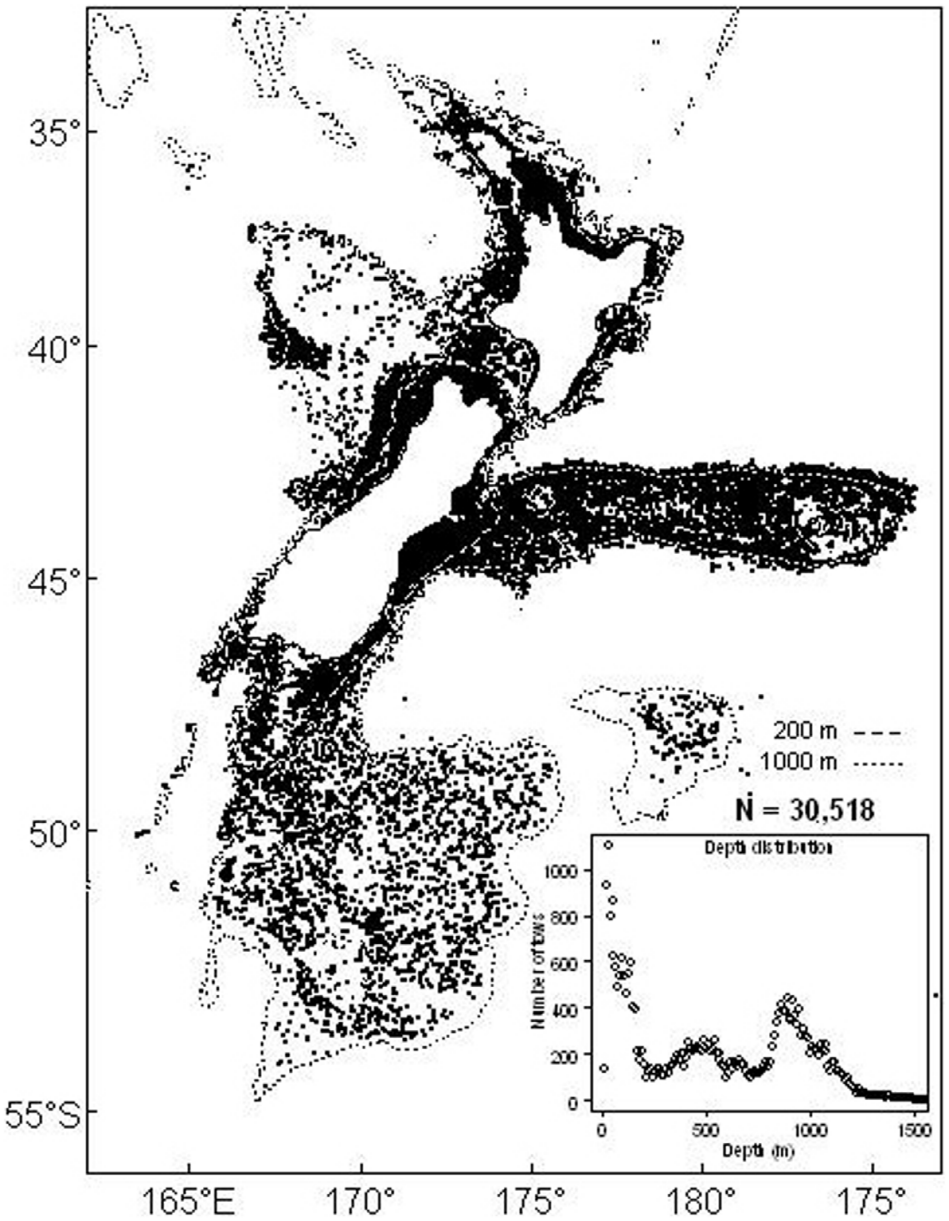
Location and depth of 30,518 bottom trawls in the NIWA Trawl Database, 1961–2009 recording the occurrences of 634 species of New Zealand fish and squid.

The National Institute of Water and Atmospheric Research (NIWA) provides by far the largest marine research activity and capability in New Zealand [25] — a position held by NIWA and its predecessor organization (mainly the New Zealand Oceanographic Institute) for more than five decades. This OBIS node currently holds about 500,000 records of benthic invertebrates and fish from the New Zealand region (and the Ross Sea). NIWA carries out the role of data manager and custodian for the fisheries research data owned by the New Zealand Ministry of Fisheries and for all seabed bathymetry data owned by Land Information New Zealand. The Fisheries Database includes Ministry of Fisheries and NIWA research trawl-survey data (30,518 records, 1961–2009: Table 4) and industry observer records (Table 5). The research trawl surveys were (and continue to be) carried out to determine demersal fish and squid distribution, abundance, population parameters, changes, and impacts on biodiversity and cover most areas of the EEZ from about 5 to 1,500 m depth (Figure 3). The data are analyzed to determine the distribution of species, the types and composition of fish communities, their changes in distribution and abundance over time, and whether these changes might be related to environmental fluctuations or fishing activity. Atlases showing the distribution of the most frequently caught demersal, midwater, and pelagic species have been published [26]–[28]. Pelagic species are also included in the database, based on aerial sightings by pilots working with purse-seine vessels. Since 1976, more than 70,700 sightings have been made from northern North Island to the mid-South Island, and data have been recorded on about 100 species including commercially important pelagics. NIWA also manages large datasets from acoustic surveys conducted between 1987 and the present around New Zealand, including echograms characteristic of specific epibenthic and midwater fish species. New Zealand's five marine laboratories, ranging in latitudinal spread from 36°16′ S to 45°49′ S, hold local marine-biodiversity data and ancillary environmental information. The oldest laboratory dates from 1951; three others opened in the early 1960s and a fifth in 2009. Two of them are adjacent to no-take marine reserves.

**Table 4:** Number of NIWA (Southwestern Pacific Regional OBIS Node) data sources for biological distributions, 1961–present.

Sampling method	No. of events
Fisheries research trawlsBottom trawlsMidwater trawls	34,010(30,711)(3,299)
Non-fish trawls	2,078
Plankton netIchthyoplanktonNonfish plankton	5,711(>3,468)*(>2,100)*
Dredge	6,219
Grab	4,703
Core	3,928
Shore collection	1,958
Sediment trap	103
Epibenthic sled	1,042
**Total**	59,752

*2000 data (method not discriminated in database).

**Table 5:** Number of Ministry of Fisheries catch events for commercial marine species in New Zealand's EEZ, October 1989–February 2009.

Fishing method	No. of events
Bottom long line	370,055
Bottom pair trawl	23,391
Beach seine/Drag net	23,673
Bottom trawl	1,411,789
Cod pots	102,936
Crab pots	13,262
Dredge	121,683
Diving	120,671
Dahn line	28,123
Inshore drift net	8,390
Dip net	64
Pair Danish seine	72
Danish seine	36,463
Fish traps	39,989
Hand gathering	70,627
Hand line	13,161
Lampara nets	1,198
Mechanical beach harvester	3,358
Midwater pair trawl	100
Midwater trawl	348,128
Octopus pots	17
Pole and line	1,130
Potting (other)	42
Purse seine	17,368
Pair set netting	3
Rock lobster pot	688,031
Ring net	32,417
Scoop net	45
Squid jig	35,700
Surface/Midwater long line	81,321
Set net (incl. gill net)	571,940
Troll	134,731
Trot line	4,973
**Total**	4,304,851

Note: The data are based on information provided in catch returns by fishers and include some events where there was effort but no catch data.

### Museums and collection data

The major marine collection in New Zealand is housed at Museum of New Zealand/Te Papa Tongarewa in Wellington. The fish collection includes 45,000 registered lots containing 195,000 specimens and 180 holotypes. The invertebrate collection comprises 490,000 lots (mostly Mollusca, with 224,000 lots) and 1,214 holotypes. The collection of marine birds is the largest in New Zealand, with about 25,000 lots. The second-largest marine collection in New Zealand is the NIWA Invertebrate Collection, Wellington, which holds specimens resulting from more than half a century of collecting in the New Zealand region, the southwestern Pacific, and the Ross Sea. The collection is arranged taxonomically and holds over 100,000 containers (not all sorted to species), representing several million specimens from more than 10,000 benthic and sediment stations, and more than 800 holotypes registered in a Specify database. Smaller collections exist at regional museums, all of which have a long history of collection and research of marine organisms. The Auckland War Memorial Museum marine collection contains specimens from all over the western Pacific and New Zealand and its offshore islands, including the Kermadecs and sub-Antarctic islands. The greater proportion is from the northern North Island, mostly from intertidal and shallow subtidal depths. There are more than 141,000 specimen lots across a range of marine phyla, including more than 90,000 molluscan lots, about 24,000 marine algal lots, nearly 7,000 crustacean lots, and 5,000 fish lots. The type collection consists of 1,108 holotypes (including 195 primary algae type sheets).

Canterbury Museum in Christchurch has about 15,000 items in the marine collection, which includes a small number of types. It has historical collections of cetaceans, inshore fish including sharks, sub-Antarctic fish, and sub-Antarctic invertebrates especially crustaceans. Otago Museum has about 24,000 items in the marine collection, of which about 160 are primary types. The largest single group is the Mollusca (about 17,000 accessioned specimens); there are also significant collections of Annelida (1,100), fishes (1,100) and Crustacea (2,500).

### Marine environmental classification

To the extent that geological, physical, chemical, and biological characteristics have been parameterized for the New Zealand region, subsets of the data have been integrated to develop a Marine Environment Classification (MEC) for the EEZ and immediately surrounding area [21]. The driver was the need for an ecosystem-based approach to marine management, necessitating a range of tools, including a classification that identifies and defines geographic areas having similar ecosystem character. Large biological datasets were used to tune the classification, and statistical tests demonstrated that the MEC classes are biologically distinctive.

### Environmental measures database

Presence-only data exported from a wide range of data sources, including NZOBIS, have been used to create Geographic Information System layers to evaluate a range of environmental measures around the coast of New Zealand [29]. The database, created for Biosecurity New Zealand to assist in the effective management of the New Zealand coastline, comprises 14 attributes of marine environmental value derived from 200 unique layers of environmental information ranging from species occurrences and diversity indices to marine-mammal breeding areas and habitat distribution. Diversity measures such as species richness, average taxonomic distinctness, species composition, and species rarity were assigned a value for each of 300 “cells” around the New Zealand coastline for each of the biological datasets. The distribution of habitats such as subtidal and intertidal rocky reef, mangrove, and seagrass was also mapped as a proportion of the total area of each habitat in the New Zealand coastline present within each cell. This has enabled the identification of areas of coincidence among high-value attributes with respect to biological and habitat diversity of the indigenous coastal marine environment.

### Alien species database

To provide a sound basis for marine biosecurity management, the New Zealand Ministry of Agriculture and Forestry Biosecurity New Zealand (MAFBNZ), between 2000 and 2008, commissioned multiple surveys of 20 New Zealand ports in addition to extensive surveys of vessel hulls [30], [31]. Following modified CRIMP (former Centre for Research on Introduced Marine Pests, CSIRO, Hobart) protocols [32]–[34], the surveys were geared towards generating baseline knowledge of overall species composition in New Zealand ports. Species identifications are coordinated through NIWA's Marine Invasives Taxonomic Service [35]. Since its inception in 2006, the MITS has logged more than 40,000 identifications comprising more than 1,100 species, of which about about 1 in 6 species are not indigenous.

### Historical data

Two historical (History of Marine Animal Populations – HMAP) projects are under way in New Zealand. The primary project, “Taking Stock,” is attempting to determine the effects of climate variation and human impact on the structure and functioning of New Zealand shelf ecosystems since first human settlement. New Zealand is interesting from a global perspective because it was the last major landmass to be settled by humans (about A.D. 1280), thus it presents a rare opportunity to examine the pattern of exploitation of marine resources in a contiguous record from earliest human settlement to the present day. All available information is being synthesized to determine the magnitude and timing of changes to the marine ecosystem and its environment over the last millennium. Modeling of the shelf ecosystem focuses on five time periods — 1,000 years ago (before human settlement), A.D. 1400 (early Māori phase), A.D. 1790 (before European sealing and whaling), 1946 (before industrial-scale fishing), and the present day. The second project is to describe the demise and slow rebuilding of whale stocks around New Zealand through the HMAP World Whaling Project, which is updating and compiling catch records from nineteenth-century whaling by American, French, British, and colonial (for example, Tasmanian) pelagic whaling fleets.

### New Zealand Inventory of Biodiversity

Over the past decade, a project has been underway to review and inventory New Zealand's entire Phanerozic biodiversity. The goal was an expert review of what is known, based on taxonomic publications, theses and “grey literature,” as well as the above-mentioned data sources, especially museum collections, to arrive at the most complete picture yet achieved, of described and known-undescribed biodiversity [36]. This project, covering all environments, all kingdoms (“bacteria to blue whales”), paleobiodiversity, Holocene extinctions, and naturalized alien species, has involved more than 220 taxonomic specialists (85 marine) in 18 countries. Outputs were to be three published volumes, now comprising one achieved, one in press and the third written but not yet sent to press [37]–[39], with the lists of living species made available to the Catalogue of Life via Species 2000 and affiliated data portals. Insofar as the species lists continue to be updated, the data compiled for this PLoS review include revised figures beyond those published in 2009 in volume 1 [37].

## Results

### Numbers of indigenous marine species

Based on expert scrutiny of the range of data sources, New Zealand has achieved a complete inventory of its known marine species, allowing its marine biodiversity to be placed in a global context for the first time, including known undescribed taxa. A list of all described New Zealand marine Animalia has been made available to OBIS as part of the Interim Register of Marine and Non-Marine Genera (http://www.obis.org.au/irmng/), to WoRMS, EoL, and the New Zealand Organisms Register for uptake.

From the tabulation compiled for this regional synthesis (Table 6, Table S1), the number of marine species including prokaryotes recorded for the New Zealand EEZ (hence within a political boundary and not the wider ecological region) is 17,135 — about 31% of the known living biodiversity of New Zealand from all environments. This figure includes 4,315 known undescribed species in collections. The best-known animal groups, with more than 1,000 species, are Mollusca with 3,593 species (2,340 described +1,253 known undescribed), 2,711 Arthropoda (2,297+414), Chordata (1,491+76), 1,435 Porifera (472+963), and 1,116 Cnidaria (794+322).

**Table 6:** Diversity of marine species found in the New Zealand region.

Taxonomic group	No. species^1^	State of knowledge	No. introduced species	No. experts	No. ID guides^2^
Domain Archaea	0	1	0	1	0
**Domain Bacteria** 3(including Cyanobacteria)	**109**	**3**	**0**	**3**	**1**
**Domain Eukarya** 3	**17,026**	**3–4**	**159**	**58**	**75**
**Kingdom Chromista** 3	**860**	**3–4**	**11**	**7**	**2**
Phaeophyta	153	4–5	11	1	1
**Kingdom Plantae** 3	**668**	**4–5**	**12**	**12**	**3**
Chlorophyta	142	3–4	0	12	1
Rhodophyta	520	3–4	12	0	1
Angiospermae	6	5	0	3	2
**Kingdom Protozoa** 3	**1,628**	**2–3**	**4**	**5**	**4**
Dinoflagellata	241	3–4	0	2	0
Foraminifera	1,076	4–5	3	2	2
**Kingdom Animalia** 3	**13,813**	**3–4**	**150**	**40**	**66**
Porifera	1,435	3	7	1	4
Cnidaria	1,116	4	23	0	6
Platyhelminthes	324	2	2	1	1
Mollusca	3,593	4	14	1	3
Annelida	791	4	32	1	2
Crustacea	2,573	3–4	27	13	17
Bryozoa	953	4	24	1	4
Echinodermata	623	5	0	3	6
Tunicata	192	5	12	1	2
Other invertebrates	646	2–5	3	7	12
Vertebrata (Pisces)	1,387	4–5	6	6	7
Other vertebrates	179	5	0	4	4
**TOTAL REGIONAL DIVERSITY** 3	**17,135**	**3–4**	**177**	**62**	**76**

1 Sources of the tallies: scientific literature, books, field guides, technical reports, museum collections.

2 Identification guides cited in Text S1.

3 Totals as reported in Table S1.

With respect to well-studied taxa, it appears that the New Zealand EEZ has high species numbers of some groups in relation to known global diversities. Among the Porifera, these include the carnivorous sponges of the family Cladorhizidae, lithistid (rock) sponges, and Hexactinellida (glass sponges). Among the Cnidaria, the Octocorallia is diversely represented by 243 species in 28 families, of which the species diversity of three deep-water gorgonian families — Chrysogorgiidae, Isididae, Primnoidae — is probably the highest in the world for a single country. Similarly, New Zealand's 66 species of Antipatharia (black corals) represent about 41% of the approximately 160 species known from the entire Indo-Pacific, and the New Zealand stylasterid (hydrocoral) fauna of 55 species is one of the most diverse in the world. Among the Mollusca, New Zealand has higher diversities of Spheniopsidae (Bivalvia) and hexactinellid-eating gastropods of the family Trochaclididae than elsewhere in the world, and the region is the global center for speciation in gastropods of the genera *Pisinna* (Anabathridae) and *Eatoniella* (Eatoniellidae). High diversities also obtain for gastropods of the families Calliostomatidae and Buccinidae. Fully 20% of the world's monoplacophorans and more than 8.5% of the world's estimated chiton species occur within the EEZ. Compared with the tropics, New Zealand's echinoderm fauna is not exceptional, but most orders are represented, the most speciose of which are the ophiuroid order Ophiurida and asteroid order Valvatida; the latter includes the crown-of-thorns sea-star, which is found at Raoul Island on the Kermadec Ridge. The New Zealand asteroid fauna includes the first-discovered species of Concentricycloidea (sea daisies), *Xyloplax medusiformis*, which was found in sunken wood from 1,100 m deep canyons off the coast of southeastern North Island and east-central South Island. At bathyal depths, New Zealand's holothurian fauna is remarkably diverse, comprising a third of the world's known species of orders Elasipodida and Molpadiida.

New Zealand's fish fauna of 1,387 species constitutes a globally unique mix of widespread (semi-cosmopolitan), Indo-Pacific, Australasian, sub-Antarctic, and endemic taxa. Approximately half the species are widespread and about 19% are endemic. The distribution of fish diversity by major habitat has been scoped for the New Zealand Marine Ecoregion and key biodiversity attributes have been identified. Currently, 148 species of cartilaginous fishes are represented in New Zealand waters. Elasmobranchs comprise a diverse group of sharks, skates, and rays, represented in New Zealand by six orders and 18 families of sharks and dogfishes, and one order and five families of skates and rays. About 90% of New Zealand's fishes are teleosts (1,233 species), in 29 orders and 185 families, of which the most diverse group is Perciformes, followed by Gadiformes, Myctophiformes, and Stomiiformes. Among families, the pelagic lanternfishes (Myctophidae), benthic and benthopelagic rattails or grenadiers (Macrouridae), and pelagic dragonfishes (Stomiidae) are the most speciose. Families that give New Zealand its unique and most distinctive character include the coastal triplefins (Tripterygiidae), clingfishes (Gobiesocidae), right-eyed flounders (Pleuronectidae), Galaxiidae, and sleepers (Eleotridae).

Out of a total avian fauna of 286 species, New Zealand has 122 species that may be classified as marine or maritime based on the criterion of spending all or almost all of their time feeding (affecting trophic and parasite/prey relationships) in those environments. Almost three quarters of the world's penguin, albatross, and petrel species and half of shearwater and shag species occur or have occurred in the New Zealand EEZ (some Holocene extinctions). Six species of penguins still nest in the EEZ and one, *Megadyptes antipodes*, represents a monotypic endemic genus. Seven of the 12 species of albatross (Diomedeidae) that currently breed in the EEZ are endemic to the New Zealand region.

Pinnipeds are important elements of New Zealand's marine ecosystems. Three species are regularly seen on the mainland and sub-Antarctic islands — the New Zealand fur seal *Arctocephalus forsteri* and, to a lesser extent and mainly in the south, the endemic New Zealand sea lion *Phocarctos hookeri* and the southern elephant seal *Mirounga leonina*.

Nearly half the world's cetaceans have been recorded in the EEZ, in other words, 43 species and subspecies. There are nine species of baleen whales, including the great whales (Balaenopteridae), such as the blue, fin, sei, Bryde's, minke, and humpback, plus the southern right whale (Balaenidae) and pygmy right whale (Neobalaenidae). There are 17 members of the dolphin family (Delphinidae), including two subspecies of endemic Hector's dolphin *Cephalorhynchus hectori*). Of the 17, 10 occur permanently and five are extralimital stragglers from tropical waters. There are 12 species of beaked whale (Ziphiidae). They are rarely seen at sea and are known mainly from stranded specimens. Three species of Physeteridae occur — sperm, pygmy sperm, and dwarf sperm whales.

There are about 1,000 species of macroalgae in the New Zealand EEZ, mostly Rhodophyta. Among these, the order Bangiales has exceptional diversity; for example there are more than 38 species of *Porphyra*, only eight of which have been named. Green macroalgae chiefly comprise members of the taxonomically complex family Ulvaceae, which includes 24 species (many unnamed) in three genera — *Gemina*, *Ulva*, *Umbraulva*. Among brown algae, the order Dictyotales is diverse in the New Zealand region with 10 genera, especially in warmer waters — 15 of the 22 native species are found on the Kermadec Islands. Likewise well represented are the Sphacelariales and Fucales. Of the five known species of the bull-kelp genus *Durvillaea*, which is restricted to the southern hemisphere, four are present in New Zealand, and three of these are endemic.

Prokaryote diversity is poorly known. There are 40 named species of blue-green “algae” (Cyanobacteria) but there have been few studies of marine bacteria in the oceans surrounding New Zealand. Isolations from inshore and coastal environments, largely from sediments, are of halophilic genera and of halotolerant genera that are also associated with terrestrial environments. Many of the genera have not yet been characterized. Isolates from oceanic water over the Chatham Rise off the east coast of New Zealand to a depth of 3,000 m have yielded representatives of halophilic *Alteromonas*, *Glaciecola*, *Halomonas*, *Oceanospirillum*, and *Pseudoalteromonas*, and halotolerant *Pseudomonas*. As part of ICoMM (International Census of Marine Microbes), microbial diversity in marine sediments of the Chatham Rise — an area of high biological activity — is being compared with that in sediments from the less productive Challenger Plateau using gene sequencing.

### Alien species

To date, 177 marine species are considered to be alien in New Zealand. This number comprises only naturalized species that have successfully bred and their propagules settled in ports and harbours, though not necessarily continuously (i.e. there may have been more than one introduction). The list excludes cryptogenic species. Among Animalia, the phyla best represented are Annelida (32 species, all but one polychaetes), Arthropoda (28, mostly malacostracan crustaceans), Bryozoa (24, mostly cheilostomes), and Cnidaria (23, mostly hydroids). Among macroalgae are 12 species of red seaweeds and 11 species of brown seaweeds.

### Spatial distribution of coastal biodiversity

**Figure 4:**
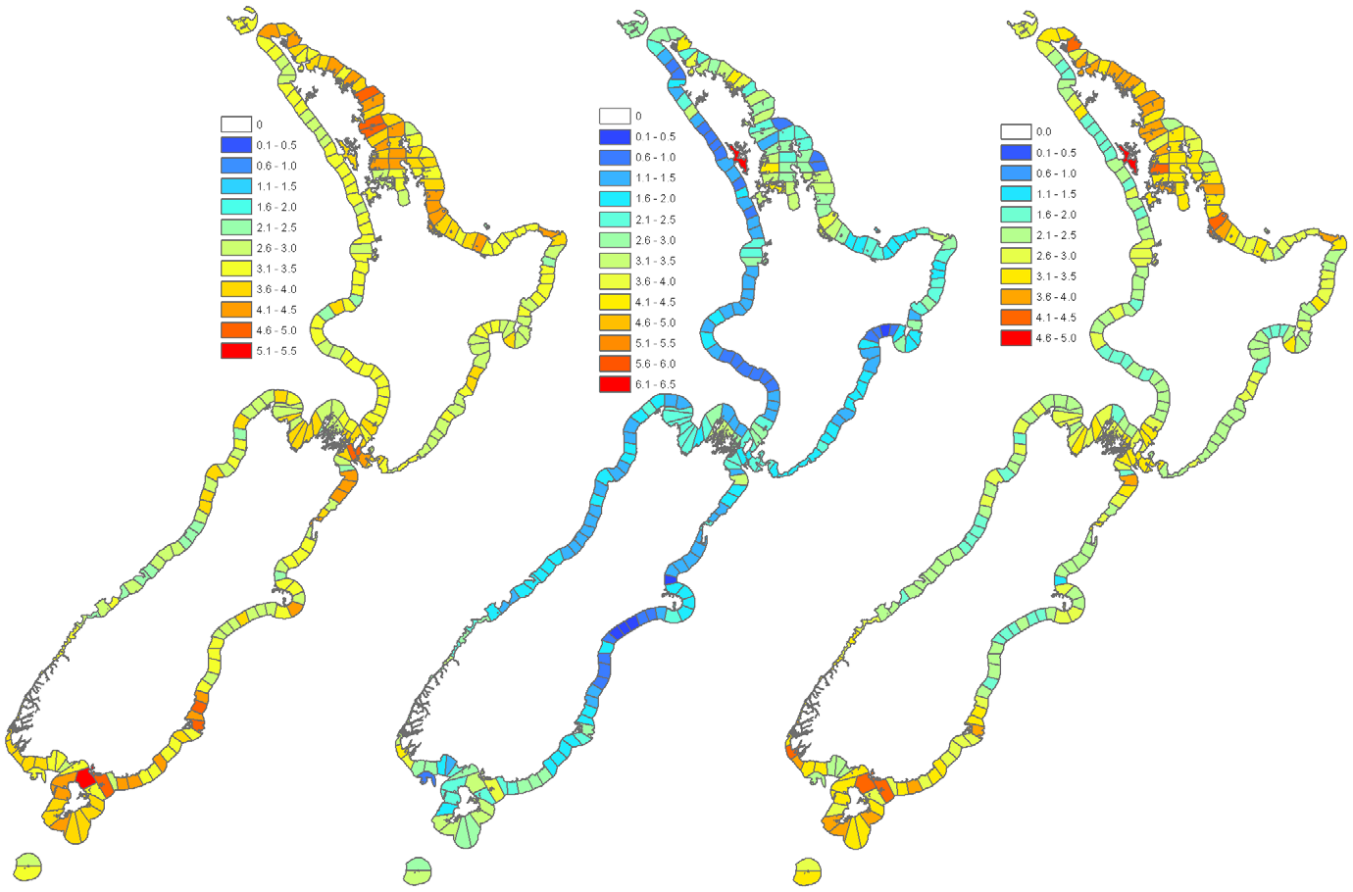
Spatial distribution of biodiversity in New Zealand's 12-nautical mile (22.2 km) territorial waters. **Left.** Spatial distribution of organismal diversity. Datasets used: taxon-specific diversity (for mollusks, echinoderms, polychaetes, bryozoans, arthropods, sponges, wading birds, diadromous fishes, rocky-reef fishes, macroalgae) at-risk and threatened birds and invertebrates, and marine-mammal distribution. **Middle.** Spatial distribution of habitat diversity. Datasets used: intertidal rocky reef, subtidal rocky reef, mangrove, seagrass, chlorophyll *a* concentration, Marine Environmental Classification (MEC) physical-habitat categories. **Right.** Overall summary map: all data (organismal and habitat combined). Values mapped for A–C are a mean rank of scores for each dataset. Hot spots are indicated by high-rank values and cold spots have low ranks. Figures reproduced from [40] by courtesy of Biosecurity New Zealand.

A first attempt to describe the spatial distribution of biodiversity around the New Zealand coastline is summarized in Figure 4 – see also [29], [40]. Synthesis maps, combining habitat and biological data, highlight many parts of the coastline as key areas for biodiversity, with several hotspots identified. The summary layers were often generated using diversity metrics derived from patchily distributed presence-only data. This is particularly true of the taxon-specific diversity datasets that contained historical/museum records, in other words, presence at a location rather than presence/absence data. As a result, generating estimates of many diversity indices was problematic, particularly so with estimates of species richness [29]; hence the summary maps are a best estimate of the coastal habitats and biodiversity around New Zealand using the available data.

## Discussion

### Analysis of known biodiversity

It is instructive to compare the New Zealand biodiversity tallies with those for the best-studied region of the world, namely, the ERMS region, which, at 21.76 million km^2^, is about five and a half times larger than the New Zealand EEZ, but has not quite twice as many species of Animalia. Interestingly, for the best-studied groups in New Zealand, the figures are comparable to those published for the entire ERMS region [41] and in some cases (Brachiopoda, Bryozoa) exceeding them (Table 7).

**Table 7:** Comparison of numbers of species of Animalia in the New Zealand EEZ and the ERMS region.

Phylum	EEZ New Zealand	ERMS Region 2001/2009 data
Porifera	1,435	**1,640/1,497**
Cnidaria	1,116	**1,316/1,515**
Mollusca	3,593	**3,798/3,844**
Brachiopoda	**50**	18/41
Bryozoa	**953**	760/769
Kinorhyncha	45	40/**51**
Echinodermata	623	**648/631**
Chordata	1,567	1,480/**1, 966**
All phyla	13,844	**25,105/25,478**

Note: New Zealand figures include described and known undescribed species [37],[38]. ERMS data are for 2001 from Costello et al. [41] and 2009 (M. Costello, pers. comm. 2010). Numbers in bold are the higher of the two regions.

The comparison is uneven insofar as the New Zealand figures include known undescribed species while the ERMS data do not, but the implication is that, when all animal phyla in New Zealand waters are as well studied, their total diversity may equal that in the very much larger ERMS region (in which the rate of discovery of new species is likely to be less than for New Zealand). Further, if this equivalence holds more or less true for all animal phyla, then the figures for the ERMS region provide a useful proxy for what kinds of species numbers may be expected in the New Zealand EEZ, regardless of whether the taxon is large or small. Major discrepancies at the phylum level pertain to Gastrotricha (only 2% of the ERMS species numbers known in New Zealand fauna and none identified to species), Tardigrada (7%), Nematoda (8%), Platyhelminthes (13%), and Nemertea (14%) owing to the paucity of collections and taxonomic work on these groups in New Zealand. Subordinate taxa with very small percentages of known species are Oligochaeta (6% of ERMS diversity), Halacaridae (marine mites) (11%), harpacticoid Copepoda (17%), and poecilostome Copepoda (18%).

### Unknown and unknowable

Expert estimates of undiscovered marine biodiversity in the New Zealand region (prokaryotes excepted) appear conservative (about 17,200 species). To a considerable extent, the figures are based on what is known about described species diversity in well-studied areas of the world, and what might thus be expected in New Zealand waters. Hence, the estimate of 2,900 species of undiscovered Protozoa in Table S1 (i.e., kingdom Protozoa as defined by Cavalier-Smith [42]) is relative to the 2,450 described protozoan species (radiolarians omitted) in the published ERMS list [41]. On the other hand, a DIVERSITAS Expert Panel, convened in 1999 to estimate the world's undiscovered biodiversity, pointed out that every well-studied metazoan species has at least one specific protozoan parasite [43]. If this is so, then there should be nearly 15,500 parasitic protozoan species for the known marine metazoans in New Zealand waters, quite apart from marine free-living Protozoa and additional parasitic Protozoa for those metazoans yet undiscovered. And how many are they? Samples taken from a 176 km transect on the continental slope of the northeastern Atlantic Ocean yielded 798 species of macroinvertebrates; extrapolations from the sample data allowed the authors to conclude that the global biodiversity of deep-sea soft sediments is on the order of 10 million species [44]. Nematodes, for example, are the most abundant multicellular organisms on earth and in marine sediments they can make up 50% to 90% of the multicellular fauna. A reconsideration of previous high estimates of global marine free-living nematode diversity led to the conclusion that about 1 million species may be realistic [45]. If so, and if 10% of this diversity (100,000 species) occurs in the New Zealand EEZ (a not unlikely percentage based on knowledge of some other taxa), then we must add to that an estimate for parasitic nematodes, protozoans, and myxozoans. Additional free-living protozoans, chromistans, fungi, plants, and metazoans will inflate this figure. In short, the number of undiscovered eukaryotes, let alone prokaryotes, in the New Zealand EEZ can only be a matter of conjecture at the present time.

Biodiversity estimates for some individual taxa or taxon groups, like fishes, on the other hand, are much more tractable. During the last 30 years, there has been intense exploration of fishing grounds by New Zealand research and commercial vessels, and improved vessels enable trawling down to 2,000 m depth. At the same time, the introduction of new collecting methods in shallow water, such as increased use of scuba and application of rotenone ichthyocide, has led to the discovery of behaviorally cryptic reef fishes. Accordingly, there has been a dramatic increase in knowledge of fish diversity such that the number of known fish species from the EEZ has doubled over the past 15 years and continues to increase at a rate of about 20 species per year, about half of which are new to science. The current estimate of undiscovered fish diversity in the EEZ is around 760 species.

### Alien species

Judging from the number of historic introductions, exotic marine species will continue to invade New Zealand coastal waters. Of the 177 naturalized species recorded to date, two out of the three of most immediate concern have entered since 2000, including the ascidian *Styela clava* and the Mediterranean fan-worm *Sabella spallanzanii* the third species, the Asian kelp *Undaria pinnatifida*, which has documented crowding and shading effects on native species) [46], was first noted in 1987.

Shipping comes to New Zealand from all around the world, but the primary trade routes involve a northerly circuit through Australia, East Asia and Southeast Asia, and a trans-Pacific circuit to the west coast of America, with the former contributing the largest volume of traffic and potential invasive species. Both of these routes include both tropical and temperate water ports, increasing the suite of potential invasive species that could be carried. Owing to the high reliance on international shipping for trade, New Zealand is vulnerable to ballast-water introductions. A significant volume of shipping traffic to New Zealand originates from East Asia, with an estimated 4.4 million tonnes of ballast water discharged in 2002 [47], the presumed vector of the invasive swimming crab *Charybdis japonica* into New Zealand [48].

Hull fouling as a dispersal vector has operated since the earliest days of shipping, and has been the most significant vector for marine species introduction to New Zealand [49], especially for encrusting species such as bryozoans, barnacles, ascidians, sponges and algae. High-speed merchant vessels like bulk carriers and container ships, with good maintenance schedules, usually have reduced levels of external fouling, but sea-chests and internal structures nevertheless consistently harbour fouling assemblages [50]. For instance, 27 percent of species in a recent survey of seachests from 42 vessels in New Zealand were Crustacea, of which at least 7 species were non-indigenous [51]. Slower-moving vessels, such as pleasure craft, some fishing vessels, barges, pontoons and oil drilling platforms present a greater risk, because of the much higher levels of fouling often present [52], [53]. Within a diverse fouling assemblage on the Maui oil platform moored off New Zealand in the 1970s, Foster and Willan [54] found half of the barnacle species to be non-indigenous. Other sources of fouling assemblages include floating wrecks and structures. Thirty-six percent of the fauna sampled from a floating wreck recently intercepted off northern New Zealand was non-native to mainland New Zealand [55].

The current state of knowledge of the number and identity of marine invasions in New Zealand is still developing. Ongoing research is required, especially in assessing relative risks associated with existing invasion pathways [56], [57]; in understanding the parameters that promote greatest propagule pressure [57]; and in fundamental taxonomic research that underpins accurate recognition of invasive and native species, e.g. [35].

### Historical trends

The HMAP project is still incomplete but results so far indicate the impact of human exploitation on selected species. For example, archeological, historical, and contemporary data have been used to estimate that over the last 700 years, some 625,000 metric tons of snapper (*Pagrus auratus*), the most important coastal food fish in the warm northern parts of New Zealand, have been removed from a large gulf on the northeast coast of North Island since the start of the fishery. Of note is that half of the total catch has been taken since 1942. Since A.D. 1300, the snapper biomass has declined 12-fold, most of this decline having occurred over the last 70 years in the largest size classes. The removal of large snapper as a predator on pelagic fishes and lesser predation on pelagic invertebrates is a major ecological consequence of the post WWII snapper fishery. Analysis of regional data on nineteenth-century whaling has been used to estimate the population trajectory of southern right whales in the New Zealand region [58]. Reconstructions suggest that right whales in New Zealand waters in about 1800, before whaling, numbered 27,000 (95% confidence interval of 22,000 and 32,000). Low estimates of minimum abundance (about 25 mature females) confirm that the population came perilously close to extinction during the late nineteenth and early twentieth centuries, while a low estimated growth rate (4.6%) suggests a rate of recovery slower than that reported for some other southern right whale breeding stocks. These results suggest that this stock was much larger than previously thought and that its ecological role in New Zealand waters as a grazer of zooplankton and as prey to killer whales and great white sharks has been underestimated.

### Threats and protections regarding New Zealand's marine biodiversity

**Figure 5:**
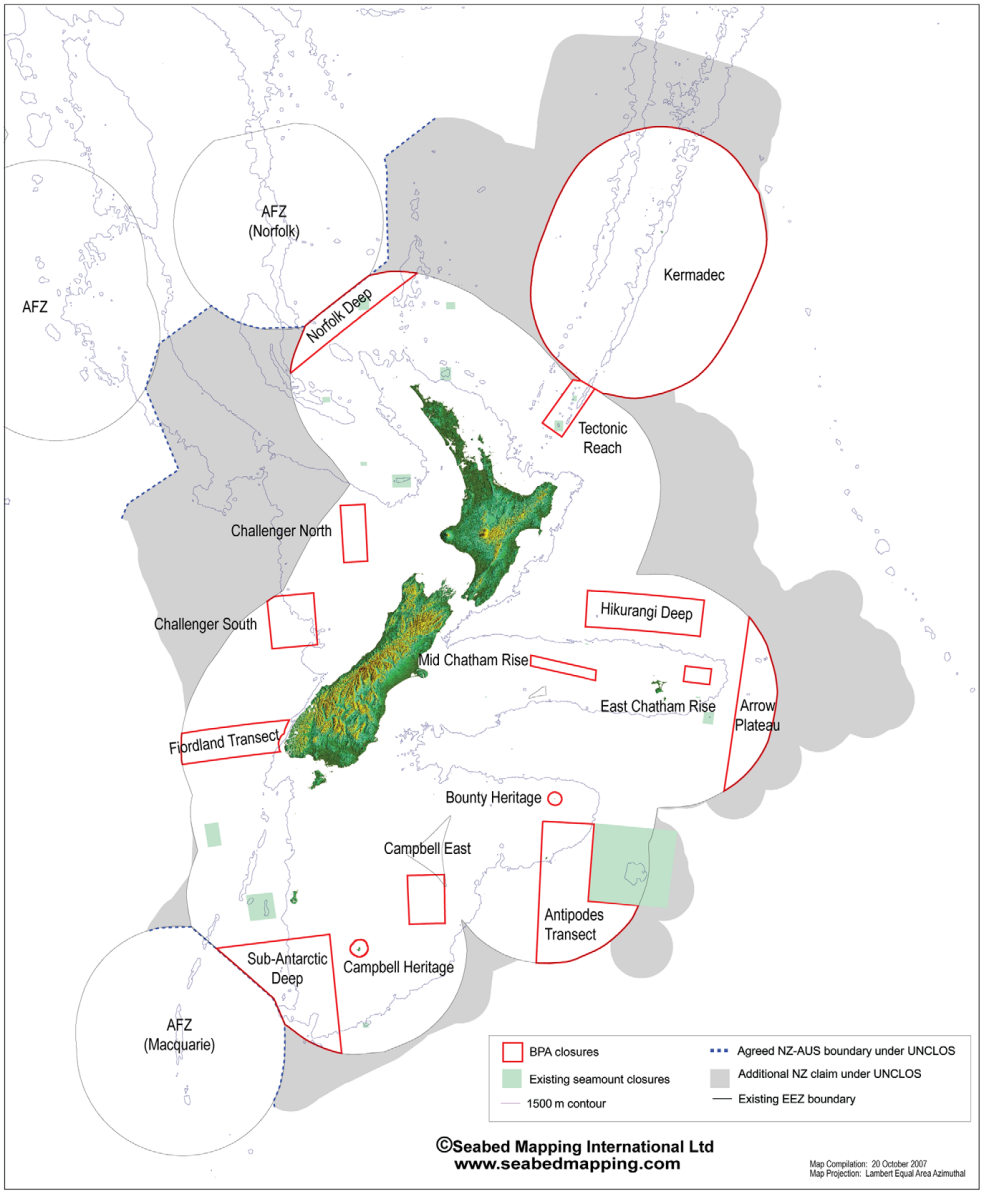
Distribution of Benthic Protection Areas, established in 2007, and seamount closures.

Given the levels of biological diversity described above, what does the future hold in store for it?What levels of protection are there and what kinds of threats? Currently, there are more than 30 marine protected areas established in New Zealand waters. All are “no take” areas, administered by the Department of Conservation (http://www.doc.govt.nz/conservation/marine-and-coastal/marine-protected-areas/marine-reserves-a-z/) [59]. They range in size from about 250 ha (within a harbor) to 745,000 ha (7,450 km^2^) (at the Kermadec Islands). Collectively, they protect 7.6% of New Zealand's territorial sea; however, 99% of this area is in two marine reserves around isolated offshore island groups (Auckland and Kermadec), and the sum of the areas of the remaining reserves in the mainland territorial sea is less than the area of the smallest terrestrial national park. Of New Zealand's total marine environment (EEZ), just 0.3% is protected in marine reserves. Currently the highest level of protection outside the territorial sea is through fisheries closures of trawling on 19 seamounts, initiated in 2001 [60]. Additionally, in 2007, the New Zealand government established 17 Benthic Protection Areas in deep water; these protect about 30% of the seabed in the EEZ from deep-sea bottom trawling and dredging activity (Figure 5). There are three marine parks, each having different regulations and generally affording a lower level of protection than marine reserves proper, for example, mainly protecting reef fish.

Māori notions of marine conservation are different from the preservationist, no-take approach. In recognition of this difference, and of Māori customary fishing rights, the Ministry of Fisheries and *iwi* (tribes) from around the country have worked to create reserves known as *mātaitai* and *taiapure* where fishing has traditional significance. Taiapure are fishing areas intended to be managed by local tribes, which recommend regulations to manage the harvest, including commercial fishing. By 2004, seven taiapure had been established. Mātaitai are areas where local tribes manage all aspects of noncommercial fishing by making bylaws, which apply to everyone. Generally, commercial fishing is not allowed within mātaitai. By 2005, mātaitai had been created at three localities.

In 1989, New Zealand's first marine mammal sanctuary was established around Banks Peninsula, as high numbers of Hector's dolphins were being caught in set nets. Set-netting is banned from November to February for 4 nautical miles in the waters around the peninsula. A second sanctuary surrounds the Auckland Islands to a distance of 12 nautical miles offshore. This protects the main breeding areas of the New Zealand sea lion and the southern right whale [61].

The threats to New Zealand's marine biodiversity are several, including fishing, mining, chemical pollution, coastal nutrient and sediment input, habitat loss, aquaculture, invasive species, harmful algal blooms, and climate change.

There is abundant literature on the environmental effects of fishing, including that in New Zealand waters [62]–[68]. Effects comprise overfishing, habitat modification or destruction, bycatch depletion, and diminishment of ecosystem services because of biodiversity loss. Impacts that have been documented in coastal waters are now mirrored in the deep sea, where recovery times tend to be much slower, owing in part to slower body metabolism and longer life histories, and often low fecundity and low recruitment. One study recorded 96 species (many undescribed) of invertebrate bycatch from a deepwater trawl fishery on the Chatham Rise [69]. Another used a photographic survey to examine coral distribution in relation to varying levels of fishing intensity in the Graveyard Seamount complex on the Chatham Rise [70]. Seamounts with a higher fishing-effects index had lower abundances and occurrence of live corals, and there were clear assemblage differences between fished and unfished seamounts in the complex [70], [71]. Smaller, faster-growing opportunistic species are favored in fished areas. The spatial extent and intensity of tows and the type of bottom-trawl gear in relation to substratum type and assemblage composition are all variables that must be taken into consideration for impact analysis.

The effects of commercial trawling — based on the large number of vessels and their mobility — are well known on soft bottoms in relatively shallow water, in which a single trawler may easily disturb 10 km^2^ in a single day's fishing, sometimes repeatedly in the same area over the course of a year, but what about in deeper water? A research study was carried out in a large New Zealand embayment (Bay of Plenty) where a burrowing deep-water species of scampi (*Metanephrops challengeri*) is taken, along with selected demersal fish, from 200 m to deeper slope depths of 600 m over an area of 2,400 km^2^. A suite of multivariate analyses revealed that fishing activity was negatively associated with invertebrate species richness and diversity and the density of several taxa, especially large, fragile, or surface-dwelling species. On the other hand, some scavenging decapod crustacean species and a sea anemone were positively associated with the fishing activity, including target scampi [72]. While soft-sediment systems of the deep sea may be enormous in extent, they are probably fragile and ill-adapted to sustain or recover from levels of disturbance commonplace in more dynamic coastal systems.

Extraction and mining of marine minerals has potential to threaten biodiversity values. Marine mineral recovery from the EEZ includes shallow-water silica sand [73], titanomagnetite ironsand, and minor aggregate (gravel), all on a relatively small scale with only localized environmental effects). Deep-water phosphorite and polymetallic manganese nodules occur in some abundance but extraction is currently economically unfeasible [74]. On the other hand, metalliferous sulphides associated with hydrothermal venting in the Kermadec arc have attracted enough interest for mining applications to be lodged and some have been granted [75]. There is some concern about the likely impact of mining on bacterial and other biodiversity insofar as many vent-associated species have very restricted distributions.

The level of marine pollution around New Zealand is low relative to that encountered in the more densely populated and industrialized countries of the northern hemisphere. A review of the information available in the early 1980s [76] makes clear that the problems encountered then remain unchanged in character today even if the quantities differ. Most oil spills in New Zealand waters are minor, but they happen reasonably frequently. Other pollutants — thermal effluent, heavy metals, and radioactivity, have been likewise relatively small in quantity (some local exceptions) and biological impact [73], [76]. New Zealand has been predominantly an agricultural economy, and some of the principal pollution problems have derived from the transfer of nutrients from animal wastes and fertilizers into coastal waters, especially those that are semi-enclosed, added to which are the contents of point discharges of municipal sewage and industrial effluent. Plastic and other litter constitute a potential hazard to marine animals and plastic items are considered to cause more deaths of marine animals than oil spills, heavy metals or other toxic materials; nevertheless, plastic debris in New Zealand waters can provide a suitable substratum for rafting and benthic species [77], [78], even in the deep sea [79].

Soil erosion, caused by the rapid development of forest clearance and agriculture over the past century, along with land-contouring for housing developments in urban areas, has led to considerable amounts of sediment and other matter entering freshwater catchments and the coastal environment. Sandy estuaries have been building up sediment at the rate of 3–6 mm per year and muddy ones at 2–5 mm per year, resulting in a 20–60 cm sediment layer over the past century. Seagrass (*Zostera muelleri*) in harbors has been one casualty, while the mangrove *Avicennia marina* has been a beneficiary. In some coastal areas, reef sponges, kelp forests, weed beds, and fish nursery grounds have been lost because of increased sediment.

Nutrient input from terrestrial runoff has been suggested as contributory to some harmful algal blooms, including red tides [80]. The majority of such blooms in New Zealand are harmless, but on several occasions potent toxins produced by a small number of harmful algal species have been reported to cause either mass mortalities of marine life. From 1978 to 2004, the eight major or “exceptional” blooms that caused fish or invertebrate mortality or toxicity to humans coincided with major El Niño/La Niña events. These blooms generally spread across large coastal areas of New Zealand and were presumably driven by fluxes of nutrients from deeper waters, through either upwelling or tidal mixing linked to the large-scale unusual weather patterns [81].

Finally, global warming and ocean acidification are expected to affect calcification in all marine organisms that have calcium carbonate skeletons, including larval and adult and free-living and sessile animals, shelled protozoans, and calcareous algae. Experimental work on dissolution of the carbonate skeleton has been carried out on nonliving New Zealand bryozoans [82].

### Potential and priorities for future discovery and research in the New Zealand region

The New Zealand Marine Sciences Society lists about 330 marine scientists nationally, of whom about 210 can be considered biologists. As can be seen from the tabulation of species diversity in Table 6 (see Results), there are currently 62 regional (in other words, New Zealand-based) scientists who are capable of identifying marine organisms. Some 29 of these are employed in universities, Crown Research Institutes (like NIWA), and museums and they regularly publish new species descriptions of marine organisms. However, funded marine-taxonomic research time for these specialists collectively totals only about 6.5 full-time equivalents per year. This is estimated to be the lowest capacity since the Second World War [83]. The balance of 33 additional scientists with identification skills includes (1) experts on marine mammals or marine angiosperms (groups in which no new species are expected or which do not require further taxonomic work), (2) taxonomists of terrestrial organisms who occasionally deal with marine forms (like fungi, or beetles and dipterans with marine larvae) and have the capacity to identify or describe them, and (3) parataxonomists who have received some training from recognized world experts in the identification of particular marine groups in the absence of local expertise.

There is significant momentum in New Zealand's marine research with main sources of funding over at least the next three to five years from the Ministry of Fisheries (including trawl surveys, environmental effects of fishing, trophic modeling, and biodiversity research); from the Foundation of Research, Science and Technology (including a wide range of research, from coastal to deep water, ecosystems modeling, habitat mapping, environmental classification, microbes to megafauna, taxonomy, biosystematics, molecular genetic sequencing, biodiversity, and biosecurity, and the effects of climate change and variability); from the Ministry of Agriculture and Forestry Biosecurity New Zealand (including baseline species surveys and targeted surveillance for unwanted marine invasive species); Universities Performance Based Research Fund (including a range of mostly smaller marine projects); and several other funding sources currently support other marine projects. Some smaller-scale projects are funded by the Department of Conservation, and also by NIWA through Capability Funds from the Foundation/Ministry of Research, Science and Technology.

Each year as a part of the Ocean Survey 20/20 program (administered by Land Information New Zealand), about 60 vessel days of the NIWA Research Vessel *Tangaroa* are funded for baseline seabed bathymetry, habitat mapping, and biodiversity sampling in a number of areas in the New Zealand EEZ and Ross Sea/Southern Ocean. Large areas have been surveyed on the Chatham Rise, Challenger Plateau (down to about 1,200 m), the Ross Sea and Southern Ocean (down to about 3,500 m), and currently in a large area of the northeastern North Island shelf out to 200 m. Although only limited funds are available for analyses from these surveys, the first two programs have already produced many new species and rich biophysical datasets, providing opportunities for exploring correlations between detailed seabed habitat maps, and associated biodiversity.

The range of hydrographic conditions and diversity of water column and benthic habitats in the New Zealand and Ross Sea areas is extensive and offers more substantial scope for research than many other oceanic regions. Exciting areas of research include questions of the scale and roles of marine viral, eubacterial, and archaebacterial microbial diversity; the role of pico-, nano-, and microphytoplankton diversity and species mix in large-scale biogeochemical processes; and the effects of ocean acidification and warming on diversity and functioning of calcareous phytoplanktonic species. Emerging research initiatives on correlations between diversity metrics and physical environmental attributes over a range of scales and the development of statistical techniques to build predictive models for small- and large-scale patterns of marine biodiversity are assisting with decisions about marine protected areas.

New and undescribed species are still common in New Zealand marine samples. Recent baseline sampling for indigenous and non-indigenous species in ports and harbors has used 10 different sampling methods and provided a surprising number of new native undescribed species — often only a few meters away from busy inner-city port structures. Recent video sequences from depths to 250 m in the southwestern South Island fiords have revealed new macroinvertebrate and fish species. Hyperbenthic sled samples from plateau and slope depths down to about 1,000 m are also rich in new species. These biodiversity discoveries indicate that more intensive sampling in commonly sampled depths will reveal more new species and, with them, more biocomplexity.

The half of the EEZ that is deeper than 2,000 m constitutes a huge area and volume down to about 10,000 m of scarcely sampled ocean. Even in depths down to 2,000 m, the shelf and slope, seamounts, undersea volcanoes, seeps, vents, canyons, and trenches offer much as-yet-unmapped topographic and chemical complexity of habitats. The scale of new-species discovery in these poorly studied or unexplored ecosystems is likely to be high for many years to come (both benthic and in the water column), although almost no research projects are likely to fund sampling in depths greater than 2,000 m.

Over the last decade, the global marine research initiatives made possible by the Census of Marine Life have increased the scale and scope of new questions and issues to be addressed in and around the New Zealand, Southwestern Pacific, and Antarctic regions. In particular, new impetus has been given to seamount biodiversity and ecology, centuries-long time-scale study of coastal ecosystems has become possible, new questions have been addressed at the level of microbial diversity, abyssal benthic sampling and use of deep-sea video imaging has opened new directions for research, and new biogeographical techniques and new syntheses are emerging.

## Supporting Information

Table S1 Diversity of described and undescribed marine species per taxon and taxonomic expertise within the New Zealand region (EEZ only). Species diversity was compiled by New Zealand and international taxonomic authorities [104-106]; subspecies are included within species binominals and are not counted separately. In the table, the names of regional experts pertain only to those living and working in the New Zealand region, not foreign experts with knowledge of the New Zealand fauna. State of knowledge: 5 =  very well-known (>90% described, identification [ID] guides <20 years old, and current taxonomic expertise); 4 =  well-known (>70% described, ID guides <50 years old, some taxonomic expertise); 3 =  poorly known (<50% species described, ID guides old or incomplete, no present expertise within region); 2 =  very poorly known (only few species recorded, no ID guides other than academic scientific papers, no expertise); 1 =  unknown (no species recorded, no ID guides, no expertise); undescr.  =  known undescribed/undetermined species; alien  =  naturalized introduced species; est'd undisc.  =  estimated undiscovered species (rounded to nearest 5 or 10 for large numbers).
(0.91 MB DOC)

Text S1 Identification guides/monographs of New Zealand marine biota.
(0.05 MB DOC)
