# Supplementary material for: Marine Biodiversity of Aotearoa New Zealand
Source: PLoS One. 2010 Aug 2;5(8):e10905. doi: 10.1371/journal.pone.0010905 (PMC2914018; doi:10.1371/journal.pone.0010905)
Supplement: Table S1 — Diversity of described and undescribed marine species per taxon and taxonomic expertise within the New Zealand region (EEZ only). Species diversity was compiled by New Zealand and international taxonomic authorities [104-106]; subspecies are included within species binominals and are not counted separately. In the table, the names of regional experts pertain only to those living and working in the New Zealand region, not foreign experts with knowledge of the New Zealand fauna. State of knowledge: 5 = very well-known (>90% described, identification [ID] guides <20 years old, and current taxonomic expertise); 4 = well-known (>70% described, ID guides <50 years old, some taxonomic expertise); 3 = poorly known (<50% species described, ID guides old or incomplete, no present expertise within region); 2 = very poorly known (only few species recorded, no ID guides other than academic scientific papers, no expertise); 1 = unknown (no species recorded, no ID guides, no expertise); undescr. = known undescribed/undetermined species; alien = naturalized introduced species; est'd undisc. = estimated undiscovered species (rounded to nearest 5 or 10 for large numbers). (0.91 MB DOC) [file pone.0010905.s001.doc]

**Supplementary Information Table S1**. Diversity of described and undescribed marine species per taxon and taxonomic expertise within the New Zealand region (EEZ only). Species diversity was compiled by New Zealand and international taxonomic authorities [104–106]; subspecies are included within species binominals and are not counted separately. In the table, the names of regional experts pertain only to those living and working in the New Zealand region, not foreign experts with knowledge of the New Zealand fauna.

State of knowledge: 5 = very well-known (>90% described, identification [ID] guides <20 years old, and current taxonomic expertise); 4 = well-known (>70% described, ID guides <50 years old, some taxonomic expertise), 3 = poorly known (< 50% species described, ID guides old or incomplete, no present expertise within region), 2 = very poorly known (only few species recorded, no ID guides other than academic scientific papers, no expertise), 1 = unknown (no species recorded, no ID guides, no expertise); undescr. = known undescribed/undetermined species; alien = naturalized introduced species; est’d undisc. = estimated undiscovered species (rounded to nearest 5 or 10 for large numbers).

| **KINGDOM**  **Phylum**  **Subphylum** | **Class**  **Subclass/**  **other category** | **Order**  **Suborder/**  **other category** | **Described species** | **State of knowledge** | **No.**  **endemic species** | **No.**  **undescr.**  **species** | **Est’d**  **undisc.**  **species** | **No. alien**  **species** | **No. regional taxonomic experts** | **No. regional ID guides**  **(totals)** |
| --- | --- | --- | --- | --- | --- | --- | --- | --- | --- | --- |
| **ANIMALIA** |  |  | **9,863** | **3–4** | **6,298** | **3,950** | **13,010**¶ | **150** | **40** | **66** |
| **Chordata** |  |  | 1,491 | 4–5 | 286 | 76 | 760 | 6 | 10 | 11 |
| Craniata |  |  | 1,490 | 4–5 | 285 | 76 | 760 | 6 | 10 | 11 |
|  | Mammalia |  | 49 | 5 | 2 | 0 | 0 | 0 | 2 | 1 |
|  |  | Cetacea | 41 | 5 | 1 | 0 | 0 | 0 | 1 | 1 |
|  |  | Carnivora | 8 | 5 | 1 | 0 | 0 | 0 | 1 | 1 |
|  |  | Sirenia | 0 | 1 | 0 | 0 | 0 | 0 | 0 | 0 |
|  | Aves |  | 122* | 5 | 41 | 2 | 0 | 0 | 2 | 3 |
|  | Reptilia |  | 6** | 5 | 0 | 0 | 0 | 0 | 0 | 0 |
|  |  | Testudines | 4** | 5 | 0 | 0 | 0 | 0 | 0 | 0 |
|  |  | Crocodylia | 0 | 1 | 0 | 0 | 0 | 0 | 0 | 0 |
|  |  | Squamata | 2** | 5 | 0 | 0 | 0 | 0 | 0 | 0 |
|  |  | Lacertilia | 0 | 1 | 0 | 0 | 0 | 0 | 0 | 0 |
|  |  | Serpentes | 2** | 5 | 0 | 0 | 0 | 0 | 0 | 0 |
|  | Myxini | Myxiniformes | 5 | 5 | 4 | 0 | 2 | 0 | 1 | 1 |
|  | Cephalaspidomorphi | Petromyzontiformes | 1 | 5 | 0 | 0 | 0 | 0 | 1 | 1 |
|  | Chondrichthyes |  | 124 | 5 | 36 | 24 | 70 | 0 | 3 | 3 |
|  | Holocephali |  | 14 | 4 | 4 | 4 | 15 | 0 | 1 | 3 |
|  | Elasmobranchii |  | 110 | 5 | 32 | 20 | 55 | 0 | 3 | 3 |
|  | Actinopterygii |  | 1,183 | 4 | 202 | 50 | 690 | 6 | 5 | 7 |
| Cephalochordata |  |  | 1 | 5 | 1 | 0 | 0 | 0 | 1 | 1 |
| **Tunicata** |  |  | 189 | 5 | 125 | 3 | 195 | 12 | 1 | 2 |
|  | Ascidiacea |  | 166 | 5 | 125 | 2 | 150 | 12 | 1 | 2 |
|  | Thaliacea |  | 18 | 5 | 0 | 1 | 24 | 0 | 0 | 0 |
|  | Larvacea |  | 5 | 5 | 0 | 0 | 22 | 0 | 0 | 0 |
| **Hemichordata** |  |  | 5 | 5 | 1 | 2 | 4 | 0 | 0 | 2 |
|  | Enteropneusta |  | 3 | 5 | 0 | 1 | 3 | 0 | 0 | 1 |
|  | Pterobranchia |  | 2 | 5 | 1 | 1 | 1 | 0 | 0 | 1 |
| **Echinodermata** |  |  | 557 | 5 | 237 | 66 | 45 | 0 | 3 | 6 |
|  | Crinoidea |  | 53 | 4 | 17 | 14 | 15 | 0 | 0 | 1 |
|  | Asteroidea |  | 170 | 5 | 113 | 18 | 5 | 0 | 1 | 5 |
|  | Ophiuroidea |  | 164 | 4–5 | 48 | 6 | 15 | 0 | 0 | 1 |
|  | Echinoidea |  | 91 | 5 | 33 | 9 | 5 | 0 | 1 | 2 |
|  | Holothuroidea |  | 79 | 5 | 27 | 19 | 5 | 0 | 1 | 2 |
| **Xenoturbellida** |  |  | 0 | 1 | 0 | 0 | 0 | 0 | 0 | 1 |
| **Platyhelminthes** |  |  | 229 | 2 | 91 | 95 | 1,810 | 2 | 1 | 1 |
| Acoelomorpha |  |  | 2 | 2 | 1 | 0 | 175 | 0 | 0 | 0 |
|  | Acoela |  | 1 | 2 | 1 | 0 | 175 | 0 | 0 | 0 |
|  | Nemertodermatida |  | 1 | 2 | 0 | 0 | 2 | 0 | 0 | 0 |
| Catenulida |  |  | 0 | 1 | 0 | 0 | 5 | 0 | 0 | 0 |
| Rhabditophora |  |  | 227 | 2 | 90 | 95 | 1,630 | 2 | 0 | 1 |
|  | Turbellaria |  | 30 | 2 | 17 | 36 | 790 | 2 | 0 | 1 |
|  | Monogenea |  | 57 | 2 | 26 | 1 | 250 | 0 | 1 | 0 |
|  | Aspidogastrea |  | 1 | 2 | 0 | 0 | 2 | 0 |  | 0 |
|  | Digenea |  | 91 | 2 | 43 | 49 | 375 | 0 |  | 0 |
|  | Cestoda |  | 48 | 2 | 4 | 9 | 215 | 0 |  | 0 |
| **Dicyemida** | Rhombozoa |  | 5 | 4 | 6 | 1 | 15 | 0 | 0 | 1 |
| **Orthonectida** |  |  | 0 | 2 | 1 | 1 | 20 | 0 | 0 | 1 |
| **Gastrotricha** |  |  | 0 | 1 | 0 | 5 | 190 | 0 | 0 | 0 |
| **Gnathostomulida** |  |  | 2 | 3 | 4 | 10 | 12 | 0 | 0 | 0 |
| **Micrognathozoa** |  |  | 0 | 1 | 0 | 0 | 1 | 0 | 0 | 0 |
| **Rotifera** |  |  | 2 | 4 | 0 | 0 | 95 | 1 | 0 | 0 |
| **Acanthocephala** |  |  | 24 | 3 | 1 | 5 | 50 | 0 | 1 | 0 |
| **Cycliophora** |  |  | 0 | 1 | 0 | 0 | 0 | 0 | 0 | 0 |
| **Mollusca** |  |  | 2,340 | 4 | 2,923 | 1,253 | 430 | 14 | 1 | 2 |
|  | Aplacophora |  | 2 | 2 | 10 | 8 | 10 | 0 |  | 0 |
|  | Polyplacophora |  | 54 | 4 | 54 | 10 | 5 | 0 |  | 2 |
|  | Monoplacophora |  | 5 | 4 | 5 | 0 | 5 | 0 |  | 0 |
|  | Gastropoda |  | 1,718 | 4 | 2,213 | 953 | 330 | 8 |  | 2 |
|  | Prosobranchia |  | 1,356 | 4 | 1,890 | 814 | 300 | 1 |  | 2 |
|  | Heterobranchia |  | 135 | 4 | 170 | 71 | 20 | 0 |  | 1 |
|  | Opisthobranchia |  | 204 | 4 | 135 | 68 | 10 | 6 |  | 2 |
|  | Pulmonata |  | 23 | 4 | 18 | 0 | 0 | 1 |  | 2 |
|  | Cephalopoda |  | 100 | 4 | 27 | 23 | 10 | 0 |  | 2 |
|  | Bivalvia |  | 450 | 4 | 569 | 223 | 62 | 6 |  | 2 |
|  | Scaphopoda |  | 11 | 4 | 45 | 36 | 10 | 0 |  | 2 |
| **Brachiopoda** |  |  | 41 | 5 | 15 | 9 | 17 | 0 | 2 | 2 |
| **Phoronida** |  |  | 3 | 4 | 0 | 0 | 2 | 0 | 0 | 1 |
| **Bryozoa** |  |  | 622 | 4 | 581 | 331 | 295 | 24 | 1 | 4 |
|  | Stenolaemata | Cyclostomata | 37 | 3 | 62 | 84 | 40 | 0 |  | 1 |
|  | Gymnolaemata |  | 585 | 4 | 519 | 247 | 255 | 24 |  | 4 |
| **Kamptozoa** |  |  | 6 | 2 | 2 | 7 | 30 | 1 | 0 | 1 |
| **Sipuncula** |  |  | 26 | 4 | 2 | 0 | 10 | 0 | 0 | 1 |
| **Annelida** |  |  | 528 | 4 | 238 | 263 | 1,005 | 32 | 1 | 2 |
|  | Polychaeta |  | 509 | 4 | 223 | 260 | 840 | 30 | 1 | 1 |
|  | Clitellata |  | 19 | 3 | 15 | 3 | 165 | 2 | 0 | 0 |
|  |  | Oligochaeta | 9 | 2 | 8 | 3 | 150 | 2 | 0 | 0 |
|  |  | Hirudinea | 11 | 4 | 7 | 0 | 15 | 0 | 0 | 0 |
| **Echiura** |  |  | 5 | 3 | 0 | 2 | 15 | 0 | 0 | 1 |
| **Nemertea** |  |  | 28 | 3 | 28 | 26 | 350 | 0 | 0 | 1 |
| **Chaetognatha** |  |  | 14 | 5 | 0 | 0 | 25 | 0 | 0 | 1 |
| **Tardigrada** |  |  | 3 | 2 | 0 | 2 | 60 | 0 | 1 | 0 |
| **Arthropoda** |  |  | 2,297 | 3–4 | 1,005 | 414 | 5,145 | 28 | 16 | 18 |
| **Chelicerata** |  |  | 115 | 3–4 | 40 | 0 | 195 | 0 | 0 | 1 |
|  | Xiphosura |  | 0 | 1 | 0 | 0 | 0 | 0 | 0 | 0 |
|  | Pycnogonida |  | 91 | 5 | 40 | 0 | 35 | 0 | 0 | 1 |
|  | Arachnida |  | 24 | 4–5 | 0 | 0 | 160 | 0 | 1 | 0 |
|  |  | Araneae | 1 | 5 | 0 | 0 | 0 | 0 | 1 | 1 |
|  | Acari | Halacaridae | 23 | 4 | 0 | 0 | 160 | 0 | 0 | 0 |
| **Myriapoda** | Diplopoda/Chilopoda |  | 0 | 1 | 0 | 0 | 0 | 0 | 0 | 0 |
| **Crustacea** |  |  | 2,166 | 3–4 | 944 | 407 | 4,930 | 27 | 11 | 17 |
|  | Branchiopoda | Cladocera | 8 | 4 | 0 | 0 | 5 | 0 | 0 | 0 |
|  | Cephalocarida |  | 1 | 5 | 1 | 0 | 1 | 0 | 1 | 1 |
|  | Remipedia |  | 0 | 1 | 0 | 0 | 0 | 0 | 0 | 0 |
|  | Maxillopoda |  | 608 | 3–4 | 123 | 120 | 1,834 | 6 | 2 | 2 |
|  | Thecostraca  (incl. Cirripedia) |  | 87 | 4 | 39 | 9 | 34 | 5 | 0 | 2 |
|  | Mystacocarida |  | 0 | 1 | 0 | 0 | 0 | 0 | 0 | 0 |
|  | Tantulocarida |  | 3 | 4 | 0 | 0 | 10 | 0 | 0 | 0 |
|  | Branchiura |  | 0 | 1 | 0 | 0 | 0 | 0 | 0 | 0 |
|  | Pentastomida |  | 0 | 0 | 0 | 0 | 0 | 0 | 0 | 0 |
|  | Copepoda |  | 518 | 3 | 84 | 111 | 1,790 | 1 | 2 | 4 |
|  | Ostracoda |  | 318 | 4 | 118 | 87 | 190 | 1 | 3 | 2 |
|  | Malacostraca |  | 1,231 | 3–4 | 702 | 200 | 2,900 | 20 | 7 | 8 |
|  |  | Leptostraca | 3 | 4 | 0 | 2 | 20 | 0 | 0 | 0 |
|  |  | Stomatopoda | 7 | 4 | 0 | 0 | 20 | 0 | 0 | 0 |
|  |  | Amphipoda | 346 | 3–4 | 226 | 52 | 550 | 12 | 3 | 1 |
|  |  | Cumacea | 48 | 4 | 67 | 26 | 90 | 0 | 0 | 1 |
|  |  | Isopoda | 269 | 3 | 254 | 66 | 1,850 | 2 | 0 | 2 |
|  |  | Mictacea | 0 | 1 | 0 | 0 | 5 | 0 | 0 | 0 |
|  |  | Mysida | 17 | 3–4 | 11 | 1 | 90 | 0 | 0 | 0 |
|  |  | Lophogastrida | 5 | 4 | 0 | 1 | 2 | 0 | 0 | 0 |
|  |  | Tanaidacea | 40 | 3 | 8 | 41 | 200 | 0 | 1 | 0 |
|  |  | Thermosbaenacea | 0 | 1 | 0 | 0 | 0 | 0 | 0 | 0 |
|  |  | Euphausiacea | 19 | 5 | 0 | 0 | 10 | 0 | 1 | 0 |
|  |  | Amphionidacea | 0 | 1 | 0 | 0 | 3 | 0 | 0 | 0 |
|  |  | Decapoda | 477 | 4 | 136 | 11 | 60 | 6 | 2 | 4 |
| **Hexapoda** | Dicondylia + Insecta |  | 16 | 3 | 21 | 7 | 20 | 1 | 3 | 0 |
| **Kinorhyncha** |  |  | 6 | 4 | 6 | 39 | 30 | 0 | 0 | 0 |
| **Loricifera** |  |  | 0 | 3–4 | 2 | 4 | 10 | 0 | 0 | 1 |
| **Priapulida** |  |  | 3 | 4 | 0 | 0 | 4 | 0 | 0 | 1 |
| **Nematoda** |  |  | 155 | 2–3 | 25 | 52 | 1,430¶ | 0 | 1 | 0 |
|  |  | free-living | 96 | 3 | 25 | 0 | >1,320¶ | 0 | 1 | 0 |
|  |  | parasitic | 23 | 2–3 | 0 | 52 | >110¶ | 0 | 0 | 0 |
| **Nematomorpha** |  |  | 1 | 5 | 1 | 0 | 0 | 0 | 0 | 1 |
| **Porifera** |  |  | 472 | 3 | 455 | 963 | 310 | 7 | 1 | 4 |
|  | Hexactinellida |  | 21 | 3–4 | 38 | 71 | 100 | 0 |  | 0 |
|  | Calcarea |  | 39 | 3 | 35 | 34 | 10 | 4 |  | 0 |
|  | Demospongiae |  | 412 | 3 | 399 | 858 | 200 | 3 |  | 4 |
| **Placozoa** |  |  | 0 | 1 | 0 | 0 | 1 | 0 | 0 | 0 |
| **Ctenophora** |  |  | 15 | 3–4 | 5 | 4 | 12 | 0 | 0 | 0 |
| **Cnidaria** |  |  | 794 | 4 | 258 | 322 | 630¶ | 23 | 0 |  |
|  | Anthozoa |  | 281 | 3–4 | 113 | 268 | 220 | 3 | 0 | 0 |
|  | Octocorallia |  | 60 | 3–4 | 40 | 183 | 75 | 0 | 0 | 0 |
|  |  | Alcyonacea  (incl. Gorgonacea) | 46 | 3 | 38 | 166 | 70 | 0 | 0 | 0 |
|  |  | Pennatulacea | 14 | 3–4 | 2 | 17 | 5 | 0 | 0 | 0 |
|  |  | Helioporacea | 0 | 1 | 0 | 0 | 0 | 0 | 0 | 0 |
|  | Hexacorallia |  | 221 | 3–4 | 73 | 85 | 145 | 3 | 0 | 0 |
|  |  | Ceriantharia | 0 | 3 | 1 | 2 | 2 | 0 | 0 | 0 |
|  |  | Actiniaria | 64 | 3–4 | 39 | 29 | 110 | 1 | 0 | 1 |
|  |  | Antipatharia | 28 | 3–4 | 14 | 38 | 10 | 0 | 0 | 0 |
|  |  | Coralliomorpha | 4 | 4 | 1 | 0 | 0 | 0 | 0 | 0 |
|  |  | Zooanthidea | 1 | 2 | 1 | 11 | 13 | 0 | 0 | 0 |
|  |  | Scleractinia | 124 | 5 | 17 | 5 | 10 | 2 | 0 | 0 |
|  | Staurozoa |  | 2 | 5 | 2 | 2 | 10 | 0 | 0 | 1 |
|  | Scyphozoa |  | 16 | 4–5 | 3 | 8 | 5 | 0 | 0 | 1 |
|  | Cubozoa |  | 1 | 5 | 0 | 0 | 2 | 0 | 0 | 1 |
|  | Hydrozoa |  | 453 | 4–5 | 122 | 28 | 253 | 20 | 0 | 3 |
|  | Trachylina |  | 34 | 5 | 1 | 1 | 13 | 0 | 0 | 2 |
|  |  | Laingiomedusae | 1 | 5 | 1 | 1 | 3 | 0 | 0 | 2 |
|  |  | Narcomedusae | 15 | 5 | 0 | 0 | 5 | 0 | 0 | 1 |
|  |  | Trachymedusae | 18 | 5 | 0 | 0 | 5 | 0 | 0 | 1 |
|  | Hydroidolina |  | 419 | 4–5 | 121 | 27 | 240 | 20 | 0 | 3 |
|  |  | Leptothecata | 234 | 5 | 77 | 14 | 130 | 10 | 0 | 2 |
|  |  | Anthoathecata | 138 | 5 | 43 | 12 | 65 | 10 | 0 | 2 |
|  |  | Siphonophora | 47 | 3–4 | 1 | 1 | 45 | 0 | 0 | 0 |
|  | Myxozoa |  | 41 | 2 | 18 | 16 | 140¶ | 0 | 0 | 1 |
| **PROTOZOA** |  |  | **1,476** | **2–3** | **162** | **152** | **2,900**¶ | **4** | **5** | **4** |
| **Foraminifera** |  |  | 961 | 4–5 | 133 | 115 | 260 | 3 | 2 | 3 |
|  | Polythalamea |  | 954 | 4–5 | 130 | 115 | 250 | 3 | B. Hayward  H. Morgans | 2 |
|  | Xenophyophorea |  | 7 | 4–5 | 3 | 0 | 10 | 0 | 0 | 1 |
| **Radiolaria†** |  |  | 165 | 4–5 | 1 | 25 | 200 | 0 | C. Hollis | 1 |
| **Myzozoa** |  |  | 238 | 3–4 | 3 | 11 | 440 | 0 | 2 | 0 |
|  | Dinozoa  (incl. dinoflagellates and *Perkinsus*) |  | 232 | 3–4 | 0 | 9 | 390 | 0 | H. Chang  A. Hayward | 0 |
|  | Apicomplexa  (incl. Sporozoa) |  | 6 | 2 | 2 | 2 | 50¶ | 0 | 0 | 0 |
| **Ciliophora** |  |  | 77 | 2 | 10 | 2 | 500¶ | 1 | 0 | 0 |
| All other Protozoa |  |  | 35 | 2 | 15 | 10 | 1,500¶ | 0 | 0 | 0 |
| **CHROMISTA** |  |  | **817** | **3–4** | **55** | **43** | **630**¶ | **11** | **7** | **2** |
| **Cryptista** | Cryptophytes, kathablepharids, etc. |  | 9 | 3–4 | 1 | 0 | 25 | 0 | H. Chang | 0 |
| **Ochrophyta** |  |  | 734 | 3–4 | 52 | 28 | 525 | 11 | 3 | 1 |
|  | Chrysophyceae |  | 14 | 3 | 0 | 4 | 110 | 0 | H. Chang | 0 |
|  | Xanthophyceae |  | 3 | 3–4 | 0 | 1 | 10 | 0 | W. Nelson | 0 |
|  | Phaeophyceae |  | 145 | 4–5 | 51 | 8 | 40 | 10 |  | 1 |
|  | Bacillariophyceae |  | 561 | 3–4 | 1 | 15 | 350 | 0 | H. Chang  M. Harper | 0 |
|  | All other classes |  | 11 | 4 | 0 | 0 | 15 | 0 | H. Chang  W. Nelson | 0 |
| **Pseudofungi** |  |  | 1 | 3–4 | 0 | 1 | 10 | 0 | 2 |  |
|  | Bigyromonadea  (*Developayella*) |  | 0 | 1 | 0 | 0 | 2 | 0 | 0 | 0 |
|  | Hyphochytrea |  | 1 | 3–4 | 0 | 0 | 6 | 0 | B. Paulus  S. Pennycook | 0 |
|  | Oomycetes |  | 0 | 3–4 | 0 | 1 | 2 | 0 |  | 1 |
| **Bigyra** |  |  | 4 | 2–3 | 0 | 5 | 35 | 0 | 1 | 0 |
|  | Actinochrysophyceae |  | 0 | 1 | 0 | 0 | 15 | 0 | 0 | 0 |
|  | Bicosoecea |  | 0 | 1 | 0 | 0 | 10 | 0 | 0 | 0 |
|  | Labyrinthulea | Thraustochytriales +  Labyrinthulales | 4 | 3 | 0 | 5 | 10 | 0 | S. Cox | 0 |
| **Haptophyta** |  |  | 68 | 4 | 2 | 9 | 15 | 0 | L. Rhodes | 0 |
| **Heliozoa††** | Centrohelida |  | 1 | 2 | 0 | 0 | 20 | 0 | 0 | 0 |
| **PLANTAE** |  |  | **567** | **4–5** | **225** | **101** | **240** | **12** | **4** | **3** |
| **Rhodophyta** |  |  | 419 | 3–4 | 189 | 101 | 150 | 12 | W. Nelson | 1 |
| **Chlorophyta** |  |  | 142 | 3–4 | 36 | 0 | 90 | 0 |  | 1 |
| **Tracheophyta** |  |  | 6 | 5 | 0 | 0 | 0 | 0 | I. Breitwieser  A. Wilton  P. Garnock-Jones | 2 |
|  |  | Mangroves | 1 | 5 | 0 | 0 | 0 | 0 |  | 1 |
| **FUNGI** |  |  | **57** | **2–3** | **0** | **0** | **210** | **0** | B. Paulus  S. Pennycook | **0** |
| **EUKARYOTA** |  |  | **12,780** | **3–4** | **6,740** | **4,246** | **16,990**¶ | **177** | **58** | **75** |
| **PROKARYOTA** |  |  | **40** | **3** | **0** | **69** | **190**¶ | **0** | **4** | **1** |
| **Archaebacteria** |  |  | 0 | 1 | 0 | 0 | –¶ | 0 | H. Morgan | 0 |
| **Cyanobacteria** |  |  | 40 | 3–4 | 0 | 2 | 190 | 0 | P. Broady | 1 |
| All other bacteria^†††^ |  |  | 0 | 3 | 0 | 67 | –¶ | 0 | E. Maas  V. Webb | 0 |
| **TOTALS** |  |  | **12,820** | **3–4** | **6,740** | **4,315** | **17,220**¶ | **177** | **62** | **76** |

* Does not include 40 additional non-nesting rare or vagrant species sighted in the New Zealand EEZ.

** Comprises only rare or vagrant species sighted in the New Zealand EEZ.

^†^ Radiolarians comprise species from two protozoan phyla ― Phaeodaria are now classified with phylum Cercozoa; the balance of radiolarians belongs to phylum Radiozoa.

**^††^** Heliozoa comprises only the Centrohelida. Actinochrysophyceae are recognized on molecular and ultrastructural grounds to belong to Bigyra; some other taxa belong to Radiozoa.

**^††^**^†^ Numbers comprise only those species for which there is at least one reference strain held in a public culture collection.

¶ These figures are highly conservative. They are estimates of the numbers of species expected to be found in New Zealand waters based on what is already described for other well-known areas of the world. For eukaryotes, realistic estimates of undiscovered species are particularly problematic for parasitic forms like myxozoans, parasitic and free- living nematodes, protozoans, and chromists; altogether, there could easily be ~40,000–50,000 undiscovered eukaryote species in New Zealand waters; for prokaryotes – who knows?
