# Supplementary material for: Marine Biodiversity of Aotearoa New Zealand
Source: PLoS One. 2010 Aug 2;5(8):e10905. doi: 10.1371/journal.pone.0010905 (PMC2914018; doi:10.1371/journal.pone.0010905)
Supplement: Text S1 — Identification guides/monographs of New Zealand marine biota. (0.05 MB DOC) [file pone.0010905.s002.doc]

**Supplementary Information Text S1.**

**Identification Guides/Monographs of New Zealand Marine Biota**

***New Zealand Oceanographic Institute Memoirs/NIWA Biodiversity Memoirs***

Annelida

Vine PJ (1977) The marine fauna of New Zealand: Spirorbinae (Polychaeta: Serpulidae). NZ Oceanogr. Inst. Mem. 68: 1–68.

Arthropoda

Barnard JL (1972) The marine fauna of New Zealand: algae-living littoral Gammaridea (Crustacea Amphipoda). NZ Oceanogr. Inst. Mem. 62: 1–216.

Bennett EW (1964) The marine fauna of New Zealand: Crustacea: Brachyura. NZ Oceanogr. Inst. Mem. 22: 1–120.

Bradford JM, Jillett JB (1980) The marine fauna of New Zealand: pelagic calanoid copepods: Family Aetidiidae. NZ Oceanogr. Inst. Mem. 86: 1–102.

Bradford JM, Haakonssen L, Jillett JB (1983) The marine fauna of New Zealand: pelagic calanoid copepods: families Euchaetidae, Phaennidae, Scolecithricidae, Diaixidae, and Tharybidae. NZ Oceanogr. Inst. Mem. 90: 1–150.

Bradford-Grieve JM (1994) The marine fauna of New Zealand: pelagic calanoid Copepoda. Families Megacalanidae, Calanidae, Paracalanidae, Mecynoceridae, Eucalanidae, Spinocalanidae, Clausocalanidae. NZ Oceanogr. Inst. Mem. 102: 1–160.

Bradford-Grieve, J.M. 1999. The marine fauna of New Zealand: pelagic calanoid Copepoda: Bathypontiidae, Arietellidae, Augaptilidae, Heterorhabdidae, Lucicutiidae, Metridinidae, Phyllopodidae, Centropagidae, Pseudodiaptomidae, Temoridae, Candaciidae, Pontellidae, Sulcanidae, Acartiidae, Tortanidae. NIWA Biodiversity Memoir 111: 1–268.

Bruce NL (2009) The marine fauna of New Zealand: Isopoda, Aegidae (Crustacea). NIWA Biodiv Mem 122: 1–252.

Child CA (1998) The marine fauna of New Zealand: Pycnogonida (sea spiders). NIWA Biodiv Mem 109: 1–71.

Forest J, de Saint Laurent M, McLaughlin PA, Lemaitre R (2000) The marine fauna of New Zealand: Paguridea (Decapoda: Anomura) exclusive of the Lithodidae. NIWA Biodiv Mem 114: 1–250.

Foster BA (1978) The marine fauna of New Zealand: barnacles (Cirripedia: Thoracica). NZ Oceanogr. Inst. Mem. 69: 1–160.

Griffin DJG (1966) The marine fauna of New Zealand: spider crabs, family Majidae (Crustacea, Brachyura). NZ Oceanogr. Inst. Mem. 35: 1–111.

Heron GA, Bradford-Grieve JM (1995) The marine fauna of New Zealand: pelagic Copepoda: Poecilostomatoida: Oncaeidae. NZ Oceanogr. Inst. Mem. 104: 1–57.

Hurley DE, Jansen KP (1977) The marine fauna of New Zealand: family Sphaeromatidae (Crustacea Isopoda: Flabellifera). NZ Oceanogr. Inst. Mem. 63: 1–95.

Jones NS (1963) The marine fauna of New Zealand: crustaceans of the order Cumacea. NZ Oceanogr. Inst. Mem. 23: 1–80.

Kornicker LS (1979) The marine fauna of New Zealand: benthic Ostracoda (suborder Myodocopina). NZ Oceanogr. Inst. Mem. 82: 1–58.

Lincoln RJ (1985) The marine fauna of New Zealand: deep-sea Isopoda Asellota, family Haploniscidae. NZ Oceanogr. Inst. Mem. 94: 1–56.

Melrose MA (1975) The marine fauna of New Zealand: family Hymenosomatidae (Crustacea, Decapoda, Brachyura). NZ Oceanogr. Inst. Mem. 34: 1–123.

Swanson KM (1979) The marine fauna of New Zealand: ostracods of the Otago Shelf. NZ Oceanogr. Inst. Mem. 8: 1–56.

Wear RG, Fielder DR (1985) The marine fauna of New Zealand: larvae of Brachyura (Crustacea, Decapoda). NZ Oceanogr. Inst. Mem. 94: 1–89.

Bryozoa

Gordon DP (1984) The marine fauna of New Zealand: Bryozoa: Gymnolaemata from the Kermadec Ridge. NZ Oceanogr. Inst. Mem. 91: 1–198 p.

Gordon DP (1986) The marine fauna of New Zealand: Bryozoa: Gymnolaemata (Ctenostomata and Cheilostomata Anasca) from the western South Island continental shelf and slope. NZ Oceanogr. Inst. Mem. 95: 1–121.

Gordon DP (1989) The marine fauna of New Zealand: Bryozoa: Gymnolaemata (Cheilostomida Ascophorina) from the western South Island continental shelf and slope. NZ Oceanogr. Inst. Mem. 97: 1–158.

Chaetognatha

Lutschinger S (1993) The marine fauna of New Zealand: Chaetognatha (arrow worms). NZ Oceanogr. Inst. Mem. 101: 1–61.

Cnidaria

Bouillon J, Barnett TJ (1999) The marine fauna of New Zealand: Hydromedusae (Cnidaria: Hydrozoa). NIWA Biodiv Mem 113: 1–136.

Cairns SD (1991) The marine fauna of New Zealand: Stylasteridae (Cnidaria: Hydroida). NZ Oceanogr. Inst. Mem. 98: 1–179.

Cairns SD (1995) The marine fauna of New Zealand: Scleractinia (Cnidaria: Anthozoa). NZ Oceanogr. Inst. Mem. 103: 1–144.

Grant RA (1976) The marine fauna of New Zealand: Isididae (Octocorallia: Gorgonacea) from New Zealand and the Antarctic. NZ Oceanogr. Inst. Mem. 66: 1–56.

Schuchert P (1996) The marine fauna of New Zealand: athecate hydroids and their medusae (Cnidaria, Hydrozoa). NZ Oceanogr. Inst. Mem. 106: 1–147.

Vervoort W, Watson JE (2003) The marine fauna of New Zealand: Leptothecata (Cnidaria: Hydrozoa) (thecate hydroids). NIWA Biodiv Mem 119: 1–538.

Echinodermata

Clark HES, McKnight DG (2000) The marine fauna of New Zealand: Echinodermata: Asteroidea (sea-stars). orders Paxillosida and Notomyotida. NIWA Biodiv Mem 116: 1–196.

Clark HES, McKnight DG (2001) The marine fauna of New Zealand: Echinodermata: Asteroidea (sea-stars) order Valvatida. NIWA Biodiv Mem 117: 1–269.

McKnight DG (2000) The marine fauna of New Zealand: Basket-stars and snake-stars (Echinodermata: Ophiuroidea: Euryalinida). NIWA Biodiv Mem 115: 1–79.

McKnight DG (1969) An outline distribution of the New Zealand shelf fauna. Benthos survey, station list, and distribution of the Echinoidea. NZ Oceanogr. Inst. Mem. 47: 1–89.

McKnight DG (2006) The marine fauna of New Zealand: Asteroidea (sea-stars). 3. Orders Velatida, Spinulosida, Forcipulatida, Brisingida with addenda to Paxillosida, Valvatida. NIWA Biodiv Mem 120: 1–187.

Pawson DL (1970) The marine fauna of New Zealand: sea cucumbers (Echinodermata: Holothuroidea). NZ Oceanogr. Inst. Mem. 52: 1–69 p.

Mollusca

Forch EC (1998) The marine fauna of New Zealand: Cephalopoda: Oegopsida: Architeuthidae (giant squid). NIWA Biodiver Mem 110: 1–113.

O’Shea S (1999) The marine fauna of New Zealand: Octopoda (Mollusca: Cephalopoda). NIWA Biodiver Mem 112: 1–280.

Nemertea

Gibson R (2002) The invertebrate fauna of New Zealand: Nemertea (ribbon worms). NIWA Biodiv Mem 118: 1–87.

Porifera

Bergquist PR (1968) The marine fauna of New Zealand: Porifera, Demospongiae. Part 1. (Tetractinomorpha and Lithistida). NZ Oceanogr. Inst. Mem. 37: 1–105.

Bergquist PR (1970) The marine fauna of New Zealand: Porifera, Demospongiae. Part 2. Axinellida and Halichondrida. NZ Oceanogr. Inst. Mem. 51: 1–85.

Bergquist PR (1996) The marine fauna of New Zealand: Porifera: Demospongiae part 5. Dendroceratida and Halisarcida. NZ Oceanogr. Inst. Mem. 107: 1–53.

Bergquist PR, Fromont PJ (1988) The marine fauna of New Zealand: Porifera, Demospongiae, Part 4 (Poecilosclerida). NZ Oceanogr. Inst. Mem. 96: 1–197.

Bergquist PR, Warne KP (1980) The marine fauna of New Zealand: Porifera, Demospongiae. Part 3. (Haplosclerida and Nepheliospongida). NZ Oceanogr. Inst. Mem. 87: 1–77.

Kelly M (2007) The marine fauna of New Zealand: Porifera: lithistid demospongiae (rock sponges). NIWA Biodiversity Memoir 121: 1–100.

Protozoa

Hedley RH, Hurdle CM, Burdett IDJ (1967) The marine fauna of New Zealand: intertidal Foraminifera of the *Corallina officinalis* zone. NZ Oceanogr. Inst. Mem. 38: 1–86.

Tunicata

Millar RH (1982) The marine fauna of New Zealand: Ascidiacea. NZ Oceanogr. Inst. Mem. 85: 1–117.

##### Other faunal/floral guides

Adams NM (1994) Seaweeds of New Zealand. Christchurch: Canterbury University Press. 360 p.

Ayling T, Cox GJ (1987) Collins guide to the sea fishes of New Zealand. Auckland: William Collins Publishers. 343 p.

Baker AN (1999) Whales and dolphins of New Zealand and Australia. An identification guide. Wellington: Victoria University Press. 133 p.

Biswell SF, compiler (2007) A fisher’s guide to New Zealand seabirds. Wellington: Department of Conservation. 55 p. Available: http://www.doc.govt.nz/upload/documents/conservation/marine-and-coastal/fishing/fishers-guide-to-nz-seabirds/fishers-guide-to-new-zealand-seabirds.pdf. Accessed 2009 Nov 4.

Cook SdeC (2009) New Zealand Coastal Marine Invertebrates 1. Christchurch: Canterbury University Press. 632 p.

Cook SdeC (In press) New Zealand Coastal Marine Invertebrates 2. Christchurch: Canterbury University Press.

Cox G, Francis M (1997) Sharks and rays of New Zealand. Christchurch: Canterbury University Press. 68 p.

Farr T, Broom J, Hart D, Neill K, Nelson W (2009) Common coralline algae of northern New Zealand: an identification guide. NIWA Inform Ser 70: 1–142.

Francis MF (2001) Coastal fishes of New Zealand – an identification guide. Revised edn. Auckland: Reed Publishing (NZ). 103 p.

Harvey A, Woelkerling W, Farr T, Neill K, Nelson W (2005) Coralline algae of central New Zealand. An identification guide to common ‘crustose’ species. NIWA Inform Ser 57: 1–145.

Hayward BW, Grenfell HR, Reid CM, Hayward KA (1999) Recent New Zealand shallow-water benthic Foraminifera: taxonomy, ecologic distribution, biogeography, and use in paleoenvironmental assessment. Inst Geol Nucl Sci Monogr 21 [NZ Geol Surv Paleo Bull 75]: vi, 1–258.

Heather BD, Robertson HA (2005) The field guide to the birds of New Zealand. Auckland: Viking Press. 432 p.

Morton JE (2004) Seashore ecology of New Zealand and the Pacific. Auckland: David Bateman. 504 p.

Paul L (2000) New Zealand fishes: identification, natural history and fisheries. Auckland: Reed Publishing (NZ). 253 p.

Paulin C, Roberts C (1993) The rock pool fishes of New Zealand, Te ika aaria o Aotearoa. Wellington: Museum of New Zealand. 177 p.

Paulin C, Stewart A, Roberts C, McMillan P (2001). New Zealand fish – a complete guide. Wellington: Te Papa Press. 279 p.

Powell AWB (1979) New Zealand Mollusca: marine, land and freshwater shells. Auckland: Collins. 500 p.

Sánchez JA (2005) Systematics of the bubblegum corals (Cnidaria: Octocorallia: Paragorgiidae) with description of new species from New Zealand and the Eastern Pacific. Zootaxa 1014: 1–72.

Sluys R, Ball IR (1988) A synopsis of the marine triclads of Australia and New Zealand. Invert Taxon 2: 915–959.

Tracey DM, Anderson OF, Naylor JR, compilers (2007) A guide to common deepsea invertebrates in New Zealand waters. Second edn. NZ Aquatic Envir Biodiv Rep 10: 1–282.
